# Supplementary material for: The aldehyde dehydrogenase 2 rs671 variant enhances amyloid β pathology
Source: Nat Commun. 2024 Mar 22;15:2594. doi: 10.1038/s41467-024-46899-0 (PMC10959958; doi:10.1038/s41467-024-46899-0)
Supplement: Supplementary file 1 — Supplementary Information [file 41467_2024_46899_MOESM1_ESM.pdf]

The aldehyde dehydrogenase 2 rs671 variant enhances amyloid β pathology

Xia Wang[1,3], Jiayu Wang[1,3], Yashuang Chen[1], Xiaojing Qian[2], Shiqi Luo[1], Xue Wang[2], Chao Ma[2,*], Wei Ge[1,*]

1 Department of Immunology, Institute of Basic Medical Sciences Chinese Academy of Medical Sciences, School of Basic Medicine Peking Union Medical College, Beijing, China.

2 Department of Human Anatomy, Histology and Embryology, Neuroscience Center, National Human Brain Bank for Development and Function, Institute of Basic Medical Sciences Chinese Academy of Medical Sciences, School of Basic Medicine Peking Union Medical College, Beijing, China.

3 These authors contributed equally: Xia Wang, Jiayu Wang.

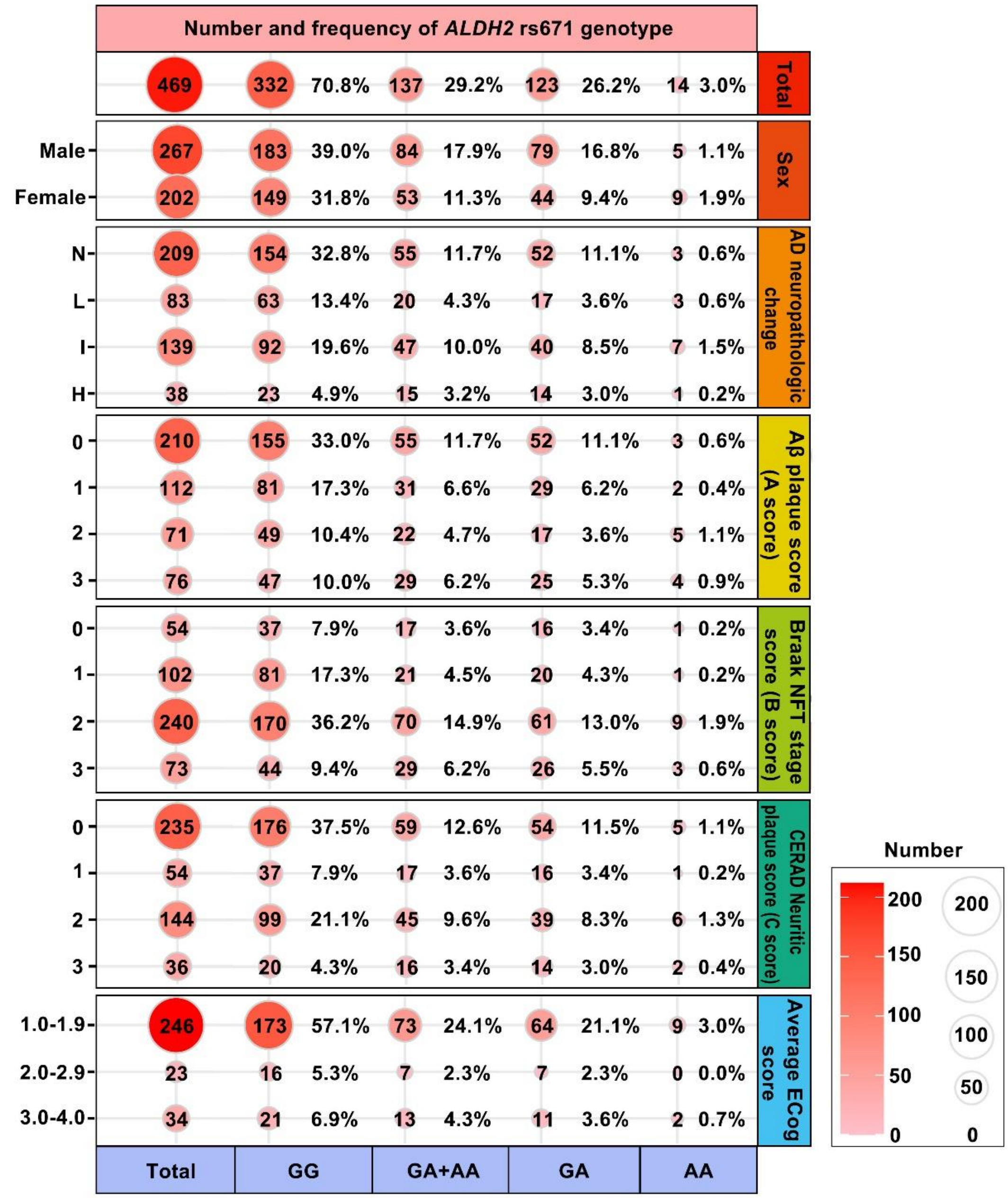

Supplementary Figure 1. The distribution of *ALDH2* rs671 genotypes and AD pathology in 469 postmortem brain donors. The AD neuropathologic change is designated as Not (N), Low (L), Intermediate (I), High (H). A score, B score, C score was evaluated according to the National Institute on Aging/Alzheimer Association as listed in Supplementary Tables 10-11.

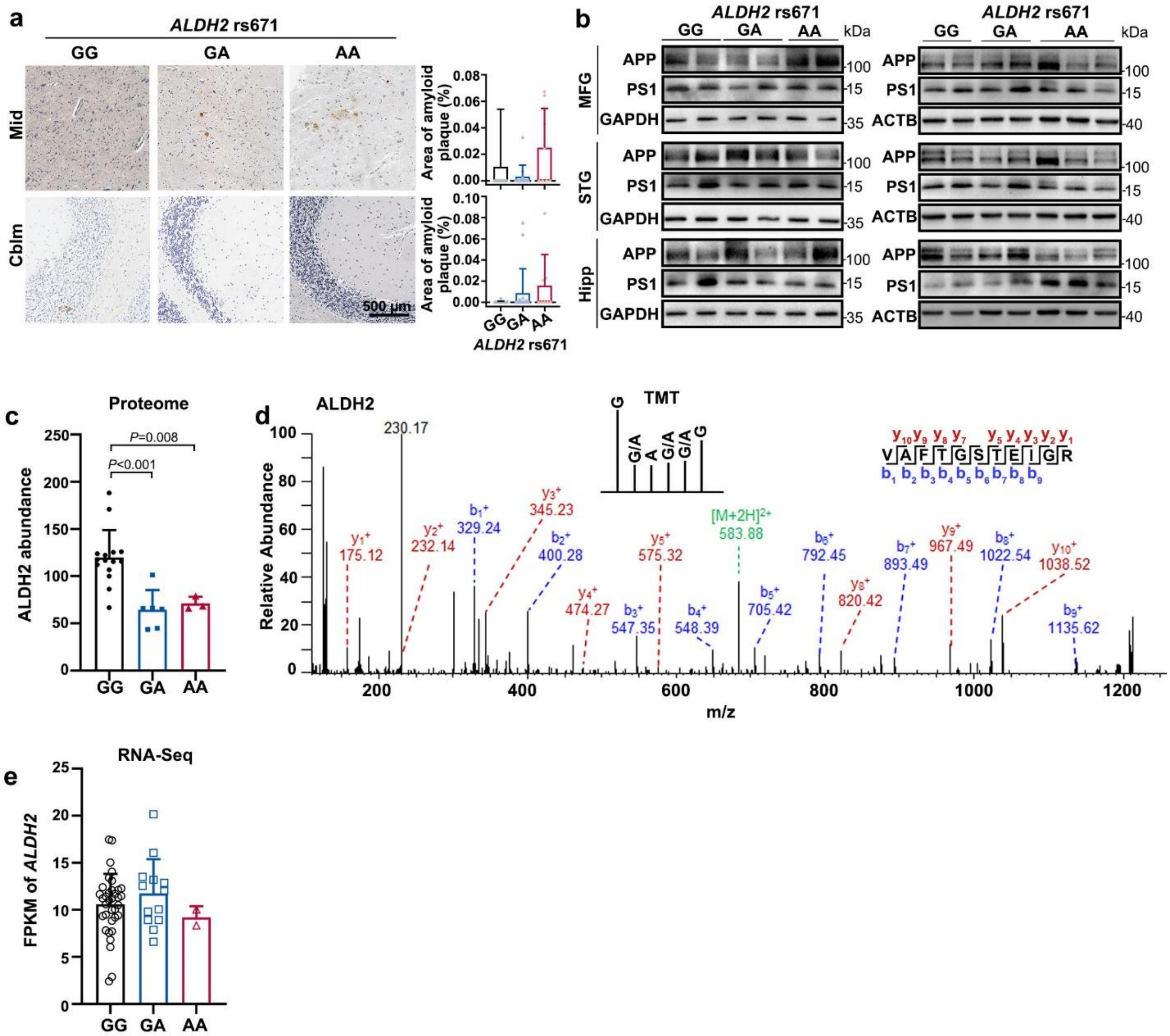

Supplementary Figure 2. Increase of Aβ plaque deposition and lower ALDH2 expression level, but not mRNA, in postmortem human brains with *ALDH2* rs671-A allele.

a, Immunohistochemical staining of amyloid plaques with 6E10 antibody in midbrain (Mid) and cerebellum (Cblm). $n = 8$ for AA genotype, $n = 18$ for GA and GG genotypes. Scale bar, 500 μm. **b**, WB detection of APP and PS1 in three brain regions (MFG, middle frontal gyrus; STG, superior temporal gyrus; Hipp, hippocampus) of pathological AD with rs671 GG ($n = 18$), GA ($n = 18$), and AA ($n = 8$) genotypes. **c**, Proteomic profiling of 24 postmortem hippocampal entorhinal cortex regions with rs671 GG ($n = 15$), GA ($n = 6$), and AA ($n = 3$) genotypes. Relative expression levels of ALDH2 are displayed with Scaled Abundance. **d**, Representative MS/MS profile of ALDH2 peptides from proteomic data of six individuals (2GG, 3GA, 1AA). **e**, RNA sequencing of 50 postmortem human hippocampal tissues with rs671 GG ($n = 35$), GA ($n = 13$), and AA ($n = 2$)

genotypes. Levels of *ALDH2* mRNA are displayed and compared with fragments per kilobase million (FPKM). Data are presented as mean values ± SD. Statistical analysis was performed using one-way ANOVA with LSD *post-hoc* test for multiple groups. Source data are provided as a Source Data file.

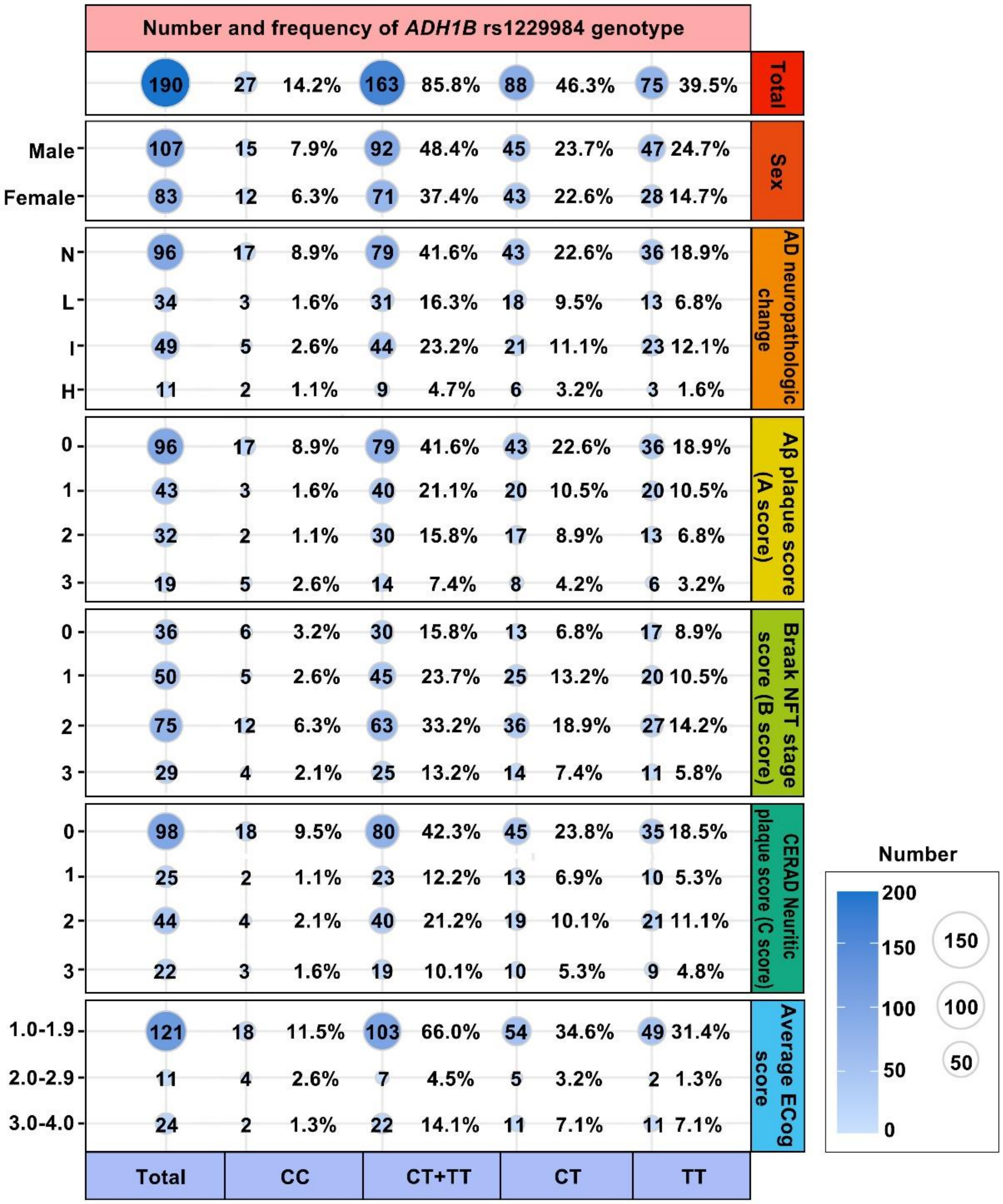

Supplementary Figure 3. The distribution of the *ADH1B* rs1229984 genotype and AD pathology in 190 postmortem brains. The AD neuropathologic change is designated as Not (N), Low (L), Intermediate (I), High (H). A score, B score, C score was evaluated according to the National Institute on Aging/Alzheimer Association as listed in Supplementary Tables 10-11.

rs1229984 genotype (*ADH1B*)		Odds ratio (95% CI)	*P* value
**AD neuropathologic change**	CC	1.00	
	CT	1.70 (0.70-4.16)	0.24
	TT	1.75 (0.71-4.36)	0.23
	CT/TT	1.73 (0.74-4.04)	0.21
**Aβ plaque score (A score)**	CC	1.00	
	CT	1.42 (0.59-3.39)	0.43
	TT	1.34 (0.55-3.27)	0.52
	CT/TT	1.38 (0.60-3.16)	0.44
**Braak NFT stage score (B score)**	CC	1.00	
	CT	1.14 (0.48-2.69)	0.77
	TT	0.80 (0.33-1.93)	0.62
	CT/TT	0.97 (0.43-2.21)	0.95
**CERAD Neuritic plaque score (C score)**	CC	1.00	
	CT	1.86 (0.74-4.65)	0.18
	TT	2.14 (0.85-5.42)	0.11
	CT/TT	1.99 (0.83-4.75)	0.12
**Ecog score**	CC	1.00	
	CT	1.18 (0.38-3.65)	0.77
	TT	1.05 (0.34-3.31)	0.93
	CT/TT	1.12 (0.39-3.22)	0.83

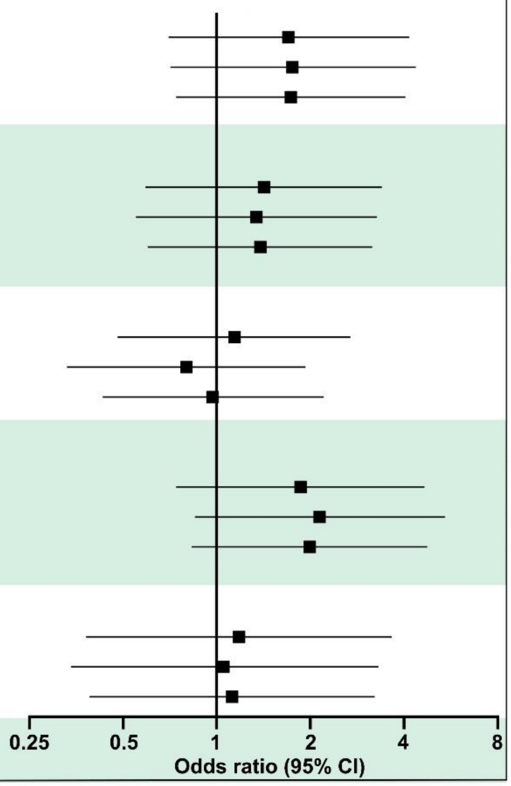

Supplementary Figure 4. Association of risk factors in populations with *ADH1B* rs1229984 polymorphism and AD neuropathologic changes after adjustment for age. Odds ratios were calculated with ordinal logistic regression using SPSS software.

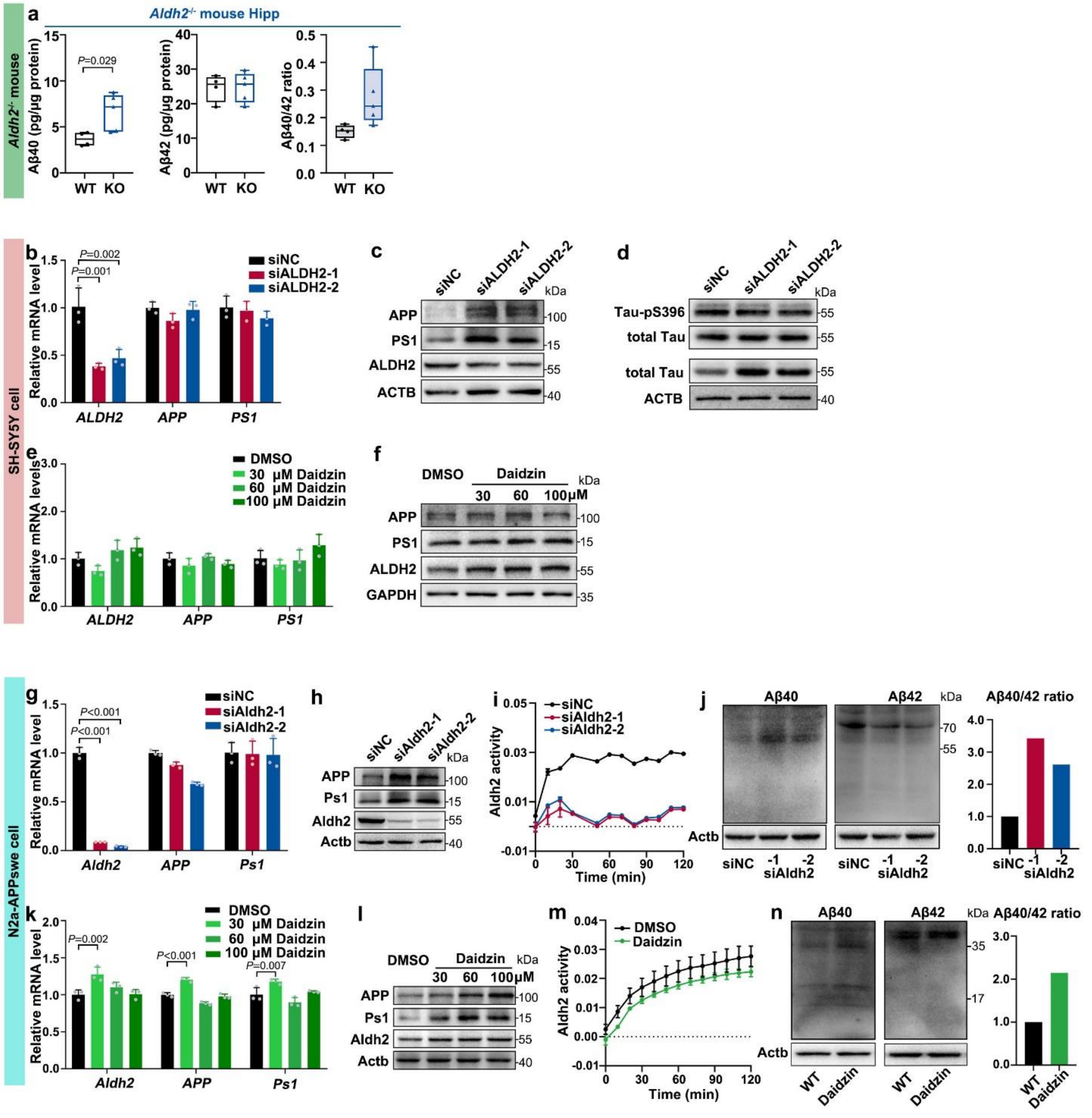

Supplementary Figure 5. ALDH2 silencing or daidzin treatment caused impaired ALDH2 enzyme activity and increased Aβ40/42 ratio in *Aldh2*-knockout mice and in multiple cells.

a, Levels of Aβ42 and Aβ40 peptides in hippocampus (Hipp) homogenates from 3-month-old *Aldh2*$^{-/-}$ mice ($n = 5$) and age-matched wild-type mice ($n = 4$) by ELISA. **b–d**, ALDH2 knockdown in SH-SY5Y cells by siRNA. (**b**) Quantitative PCR (n=3 biologically independent samples) and (**c**) WB detection of ALDH2, APP, and PS1 mRNA and protein expression levels. (**d**) WB detection of total Tau and Tau-pS396 proteins. **e–f**, SH-SY5Y cells treated with different concentrations of daidzin (30–100 μM) for 48 h. (**e**) Quantitative PCR (n=3 biologically independent samples) and

(**f**) WB detection of ALDH2, APP, and PS1 mRNA and protein expression levels. **g–j**, N2a-APPswe cells with Aldh2 knockdown. (**g**) Quantitative PCR (*n*=3 biologically independent samples) and (**h**) WB detection of Aldh2, APP, and Ps1 mRNA and protein expression levels. (**i**) Enzymatic activity of Aldh2 in cell lysates measured over 120 min. *n*=3 biologically independent samples. (**j**) WB detection of Aβ40 and Aβ42. **k–n**, N2a-APPswe cells with different concentrations of daidzin treatment (30–100 μM) for 48 h. (**k**) Quantitative PCR (*n*=3 biologically independent samples) and (**l**) WB detection of Aldh2, APP, and Ps1 mRNA and protein expression levels. (**m**) Enzymatic activity of Aldh2 (*n*=3 biologically independent samples) and (**n**) WB detection of Aβ40 and Aβ42 in cell lysates pretreated with 60 μM daidzin for 48 h. Data are presented as mean values ± SD. All box plots include the median line, the box indicates the interquartile range, whiskers indicate minima and maxima. Statistical analysis was performed using two-tailed Student's *t*-test. Source data are provided as a Source Data file.

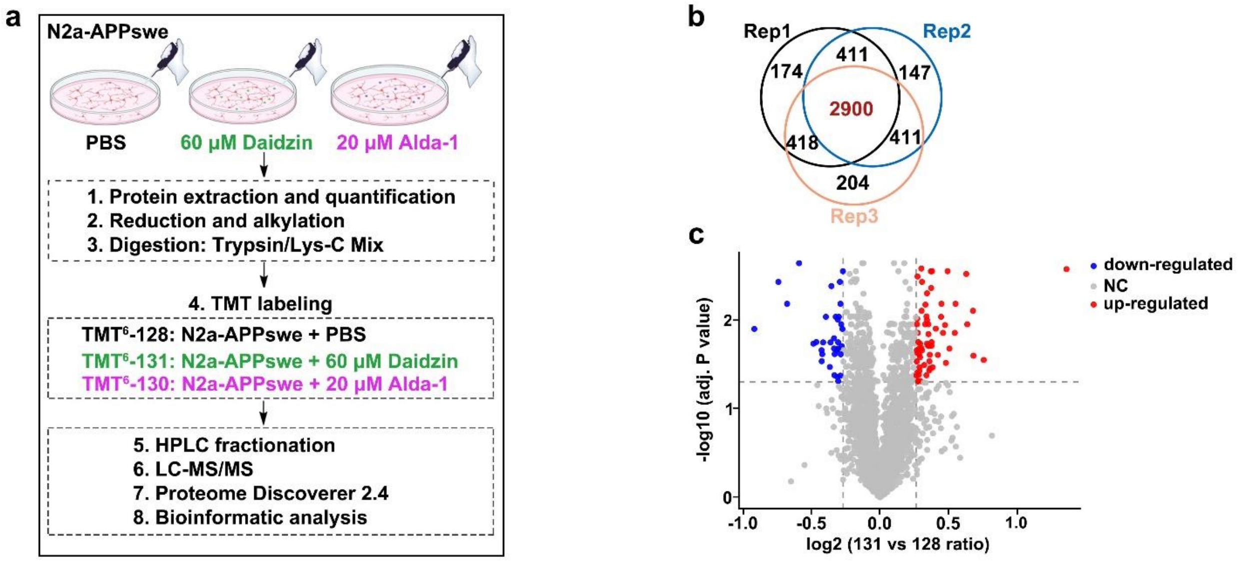

Supplementary Figure 6. Proteomic analysis of N2a-APPswe cells pretreated with 60 μM daidzin for 48 h or 20 μM Alda-1 for 24h.

a, Flowchart of the proteomic study design. **b**, Venn map of confident proteins detected in three replicates. c, Volcano plot of distributions of confident proteins. The threshold for differentially expressed proteins screening was set as: adjusted *P* value (BH corrected) ≥ 0.05, fold change ≤ 0.83 or ≥ 1.20. Image was created using elements from ChemBioDraw Ultra software 14.0. 128, N2a-APPswe + PBS, 131, N2a-APPswe + daidzin, 130, N2a-APPswe + Alda-1.

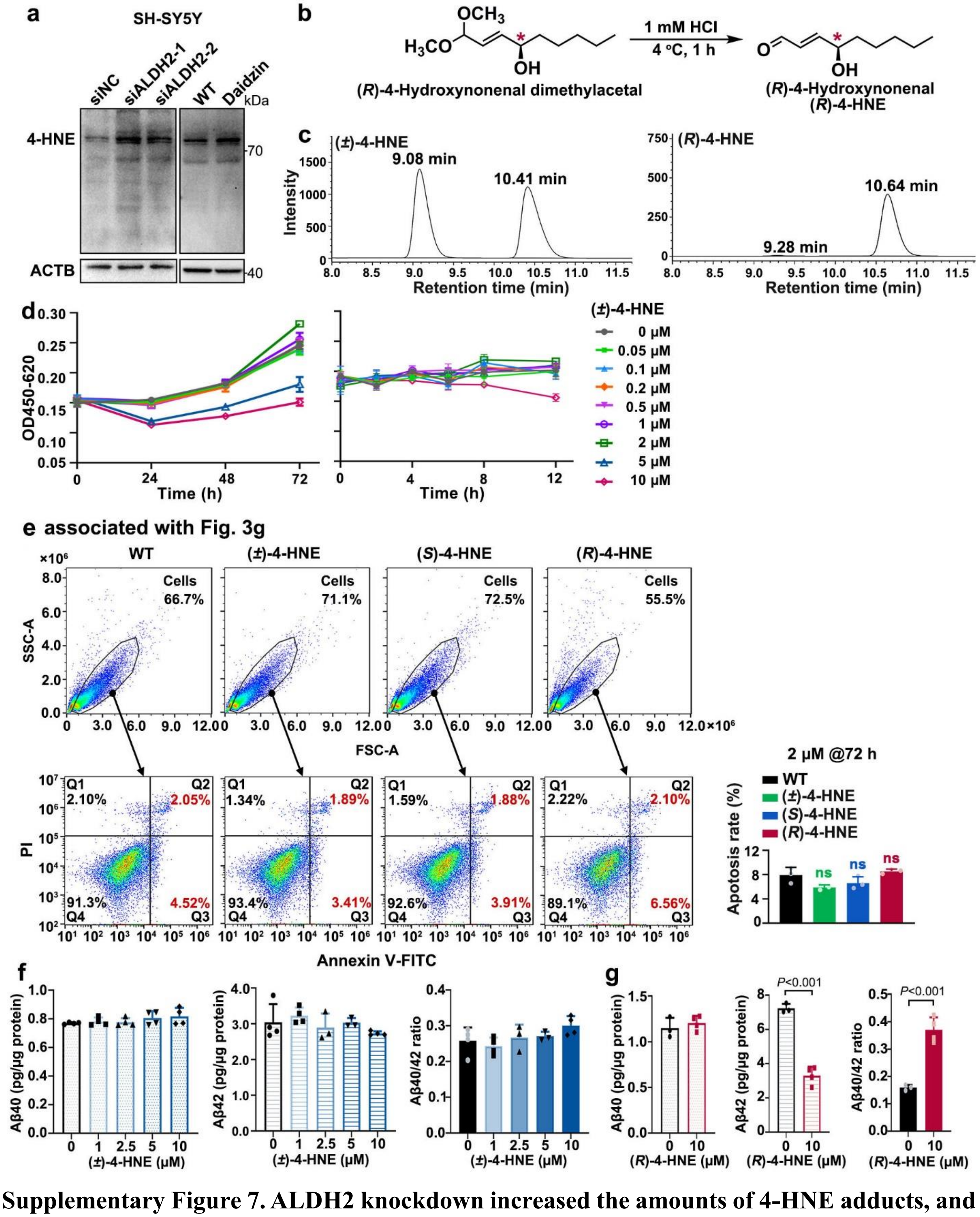

Supplementary Figure 7. ALDH2 knockdown increased the amounts of 4-HNE adducts, and (*R*)-4-HNE increased the Aβ40/42 ratio, in SH-SY5Y cells.

a, WB detection of 4-HNE-adducted proteins in ALDH2-deficient SH-SY5Y cells derived from ALDH2 silencing or 60 μM daidzin treatment for 48 h. **b**, Generation of (*R*)-4-HNE from precursor

(*R*)-4-hydroxynonenal dimethylacetal. **c**, Analysis of racemic (±)-4-HNE, and standard (*R*)-4-HNE derived from precursor, using a chiral HPLC column (CHIRALPAK AS-H, Japan). **d**, Proliferation of SH-SY5Y cells pretreated with different concentrations of (±)-4-HNE for varied incubation times (0–72 h). *n*=3 biologically independent samples. **e**, The flow-cytometry gating strategies were shown (left). Statistics for flow cytometry analysis of apoptosis rates in SH-SY5Y cells pretreated with (±)-4-HNE, (*R*)-4-HNE, or (*S*)-4-HNE at 2 μM for 72 h (right), associated with **Fig. 3g**. *n*=3 biologically independent samples. **f–g**, Levels of intracellular Aβ40 and Aβ42 determined by ELISA in SH-SY5Y cells (**f**) pretreated with 0, 1, 2.5, 5, or 10 μM (±)-4-HNE for 4 h, or (**g**) pretreated with 10 μM (*R*)-4-HNE for 4 h. *n*=4 biologically independent samples. Data are presented as mean values ± SD. Statistical analysis was performed using two-tailed Student's *t*-test for two groups and one-way ANOVA with LSD *post-hoc* test for multiple groups. Source data are provided as a Source Data file.

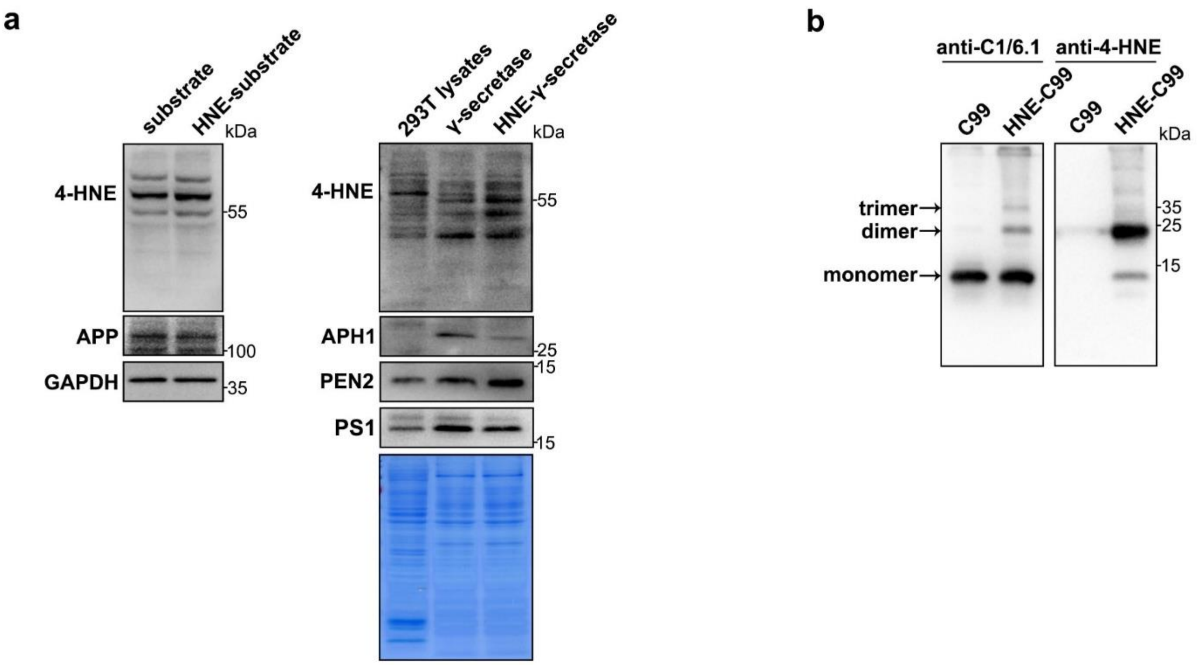

Supplementary Figure 8. WB analysis of substrates and γ-secretase in *in vitro* γ-secretase cleavage assays.

a, WB of substrates from N2a-APPswe cells, WB and coomassie brilliant blue staining of γ-secretase complex from HEK293T cells. **b**, WB of recombinant C99 and 4-HNE-adducted C99. Source data are provided as a Source Data file.

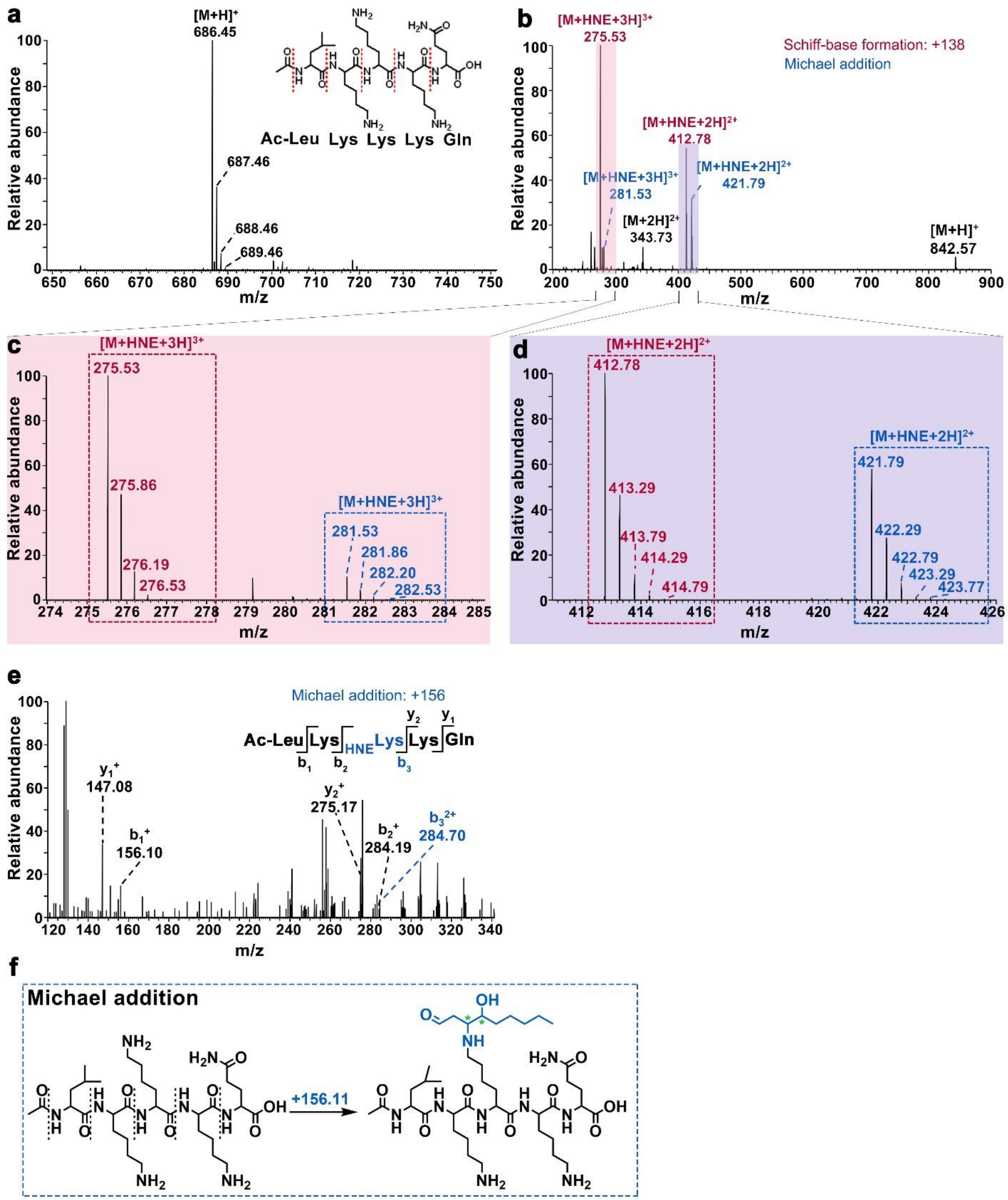

Supplementary Figure 9. Mass spectrometry of peptide Ac-Leu–Lys–Lys–Lys–Gln and 4-HNE-modified peptides.

a, MS of peptide Ac-Leu–Lys–Lys–Lys–Gln (residues 52-56 of C99). **b**, MS of 4-HNE-modified peptides. Red represents the major product, formed via Schiff-base formation; blue represents a

lower-yield product formed via Michael addition. **c–d**, Enlargements of (**b**). **e**, Representative MS/MS profile of Ac-Leu–Lys–Lys(HNE)–Lys–Gln peptide containing the second Lys residue (Lys54 of C99) modified by 4-HNE via Michael addition. **f**, Michael addition reaction of Ac-Leu–Lys–Lys–Lys–Gln with 4-HNE to generate Ac-Leu–Lys–Lys(HNE)–Lys–Gln.

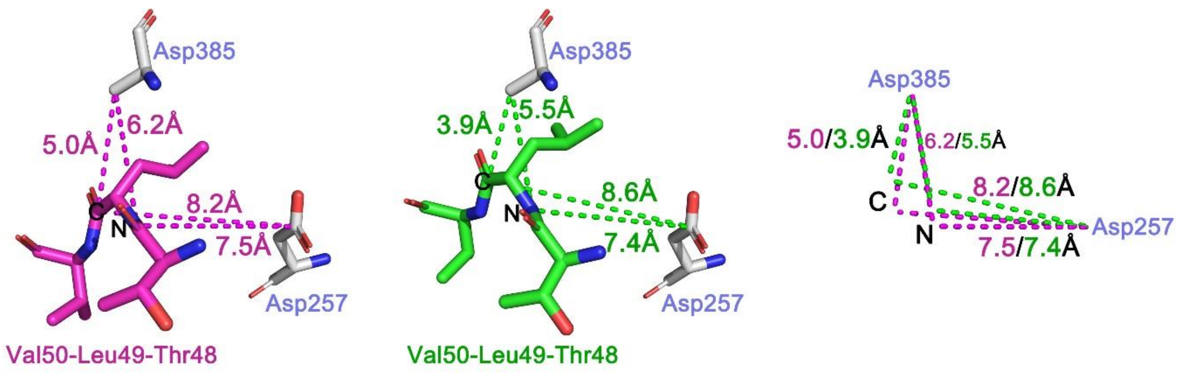

Supplementary Figure 10. The predicted distance between the carboxylate side chain of Asp385 or Asp257 from PS1 and the C=O group or NH group of Leu49 from C99. The natural C99 residues (purple) and the 4-HNE modified C99 residues (green).

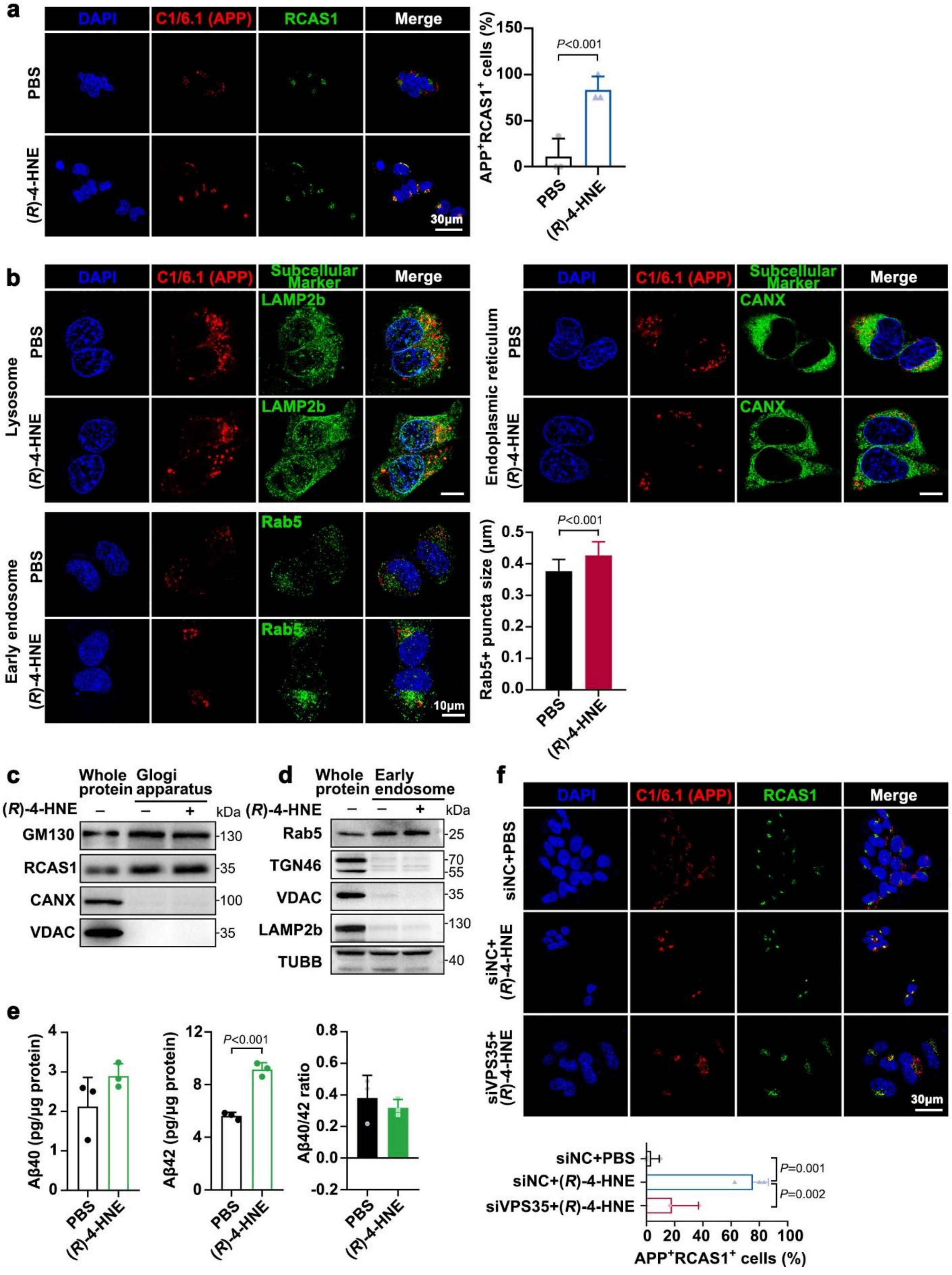

Supplementary Figure 11. (*R*)-4-HNE did not affect Aβ40/42 ratio in early endosomes.

a-b, Representative images of HEK293T cells treated with 2 μM (*R*)-4-HNE for 24 h and co-stained for APP (red, C1/6.1) and subcellular markers (green, RCAS1 for golgi, Lamp2b for lysosomes, CANX for the endoplasmic reticulum, and Rab5 for early endosome). 4′,6-Diamidino-2-phenylindole (DAPI; blue) was used as a nuclear counterstain. *n*=3 biologically independent samples. Scale bar, 30 μm (**a**). Scale bar, 10 μm (**b**). And quantification of Rab5^{+} puncta size by using Image Pro Plus software. **c,** Golgi apparatus was isolated from HEK293T cells pretreated with PBS or (*R*)-4-HNE, and verified by WB with specific Golgi apparatus markers (GM130, RCAS1) and other organelle markers (CANX for the endoplasmic reticulum, VDAC for mitochondria). **d–e**, HEK293T cells were treated with 2 μM (*R*)-4-HNE for 24 h. Early endosomes were isolated and enriched by using an Endosome Isolation and Cell Fractionation Kit (Invent, ED-028). (**d**) WB with specific early endosome marker (Rab5) and other organelle markers (TGN46 for Golgi apparatus, VDAC for mitochondria, LAMP2b for lysosome). (**e**) Levels of Aβ40 and Aβ42 in early endosome lysates. *n*=3 biologically independent samples. **f**, Representative images of co-staining for APP (red, C1/6.1) and RCAS1 (green, Golgi) in VPS35 knockdown HEK293T cells and relative siNC cells. *n*=3 biologically independent samples. Data are presented as mean values ± SD. Statistical analysis was performed using two-tailed Student's *t*-test for two groups. Source data are provided as a Source Data file.

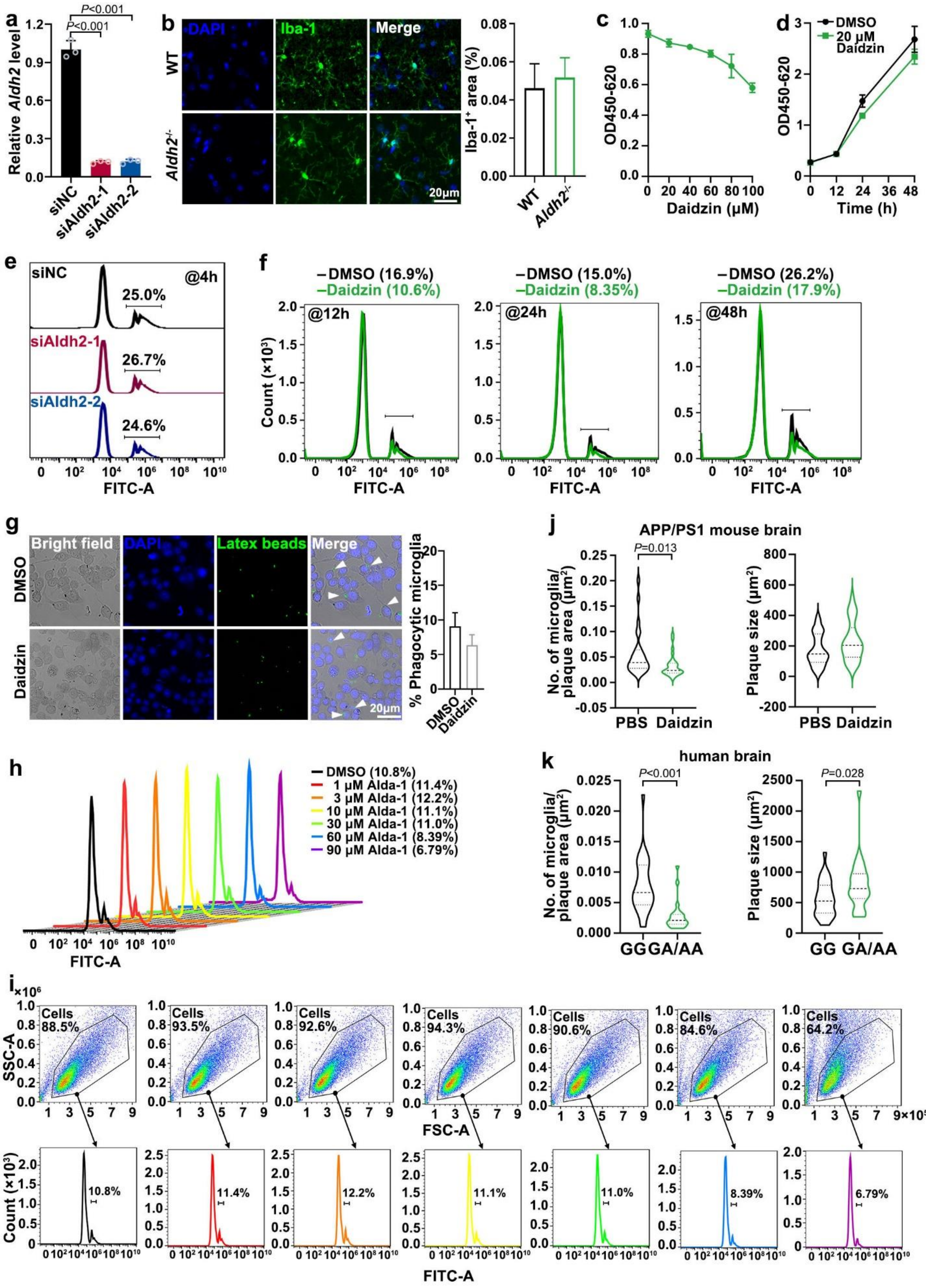

a
Relative Aldh2 level
P<0.001
P<0.001
siNC
siAldh2-1
siAldh2-2
b
DAPI
Iba-1
Merge
WT
Aldh2-/-
20μm
Iba-1+ area (%)
c
OD450-620
Daidzin (μM)
d
DMSO
20 μM Daidzin
Time (h)
e
siNC
@4h
25.0%
siAldh2-1
26.7%
siAldh2-2
24.6%
FITC-A
f
DMSO (16.9%)
Daidzin (10.6%)
@12h
DMSO (15.0%)
Daidzin (8.35%)
@24h
DMSO (26.2%)
Daidzin (17.9%)
@48h
Count (×10³)
FITC-A
g
Bright field
DAPI
Latex beads
Merge
DMSO
Daidzin
20μm
% Phagocytic microglia
h
DMSO (10.8%)
1 μM Alda-1 (11.4%)
3 μM Alda-1 (12.2%)
10 μM Alda-1 (11.1%)
30 μM Alda-1 (11.0%)
60 μM Alda-1 (8.39%)
90 μM Alda-1 (6.79%)
FITC-A
i
Cells 88.5%
Cells 93.5%
Cells 92.6%
Cells 94.3%
Cells 90.6%
Cells 84.6%
Cells 64.2%
SSC-A
FSC-A
10.8%
11.4%
12.2%
11.1%
11.0%
8.39%
6.79%
Count (×10³)
FITC-A
j
APP/PS1 mouse brain
P=0.013
No. of microglia/ plaque area (μm²)
Plaque size (μm²)
PBS
Daidzin
k
human brain
P<0.001
P=0.028
No. of microglia/ plaque area (μm²)
Plaque size (μm²)
GG
GA/AA

Supplementary Figure 12. Decreasing ALDH2 activity suppressed Aβ-induced microglial activation and phagocytic phenotype.

a, Quantitative PCR detection of *Aldh2* silencing in BV2 cells. *n*=3 biologically independent samples. **b**, Representative images of microglia (stained with Iba-1) in the cortex from 3-month-old *Aldh2*$^{-/-}$ and age-matched C57BL/6 mice. Quantification of microglial cell density in the cortex. *n* = 3 mice per group; five fields (4×10^4 μm^2 per field) of view were randomly selected from each mouse for statistical analysis. Scale bar, 20 μm. **c–d**, Proliferation of BV2 cells treated (**c**) with different concentrations of daidzin (20, 40, 60, 80, and 100 μM) for 6 h (*n* = 3–4), and (**d**) with 20 μM daidzin for different incubation times (12, 24, and 48 h, *n* = 6–8). **e–f**, Flow cytometry histogram showing the phagocytosis rate of latex beads of BV2 cells with (**e**) Aldh2 silencing or (**f**) stimulated with daidzin (20 μM) for 12, 24 and 48 h. The flow-cytometry gating strategies were shown in **Supplementary Figure 13a and b**, respectively. **g**, Representative images of BV2 cells co-incubated with 20 μM daidzin and latex beads for 6 h. Five fields of view were randomly selected from each group for statistical analysis. Two independent biological replicates were performed. Scale bar, 20 μm. **h**, Flow cytometry histogram showing the phagocytosis rate of BV2 cells treated with Alda-1, an ALDH2 activator, at different concentrations (0, 1, 3, 10, 30, 60, and 90 μM) for 2 h. **i**, The flow cytometry gating strategies of (**h**). **j**, Quantification of microglial cells in the cortex from APP/PS1 and daidzin-treated APP/PS1 mice within 20 μm of a plaque surface (*n* = 28 plaques from 3 mice per group). Associated with **Fig. 7f.** **k**, Quantification of microglial cells in the middle frontal gyrus from patients with AD within 20 μm of plaque surface (*n* = 24–28 plaques from 3 patients with AD per group). Associated with **Fig. 7g.** Data are presented as mean values ± SD. Statistical analysis was performed using two-tailed Student's *t*-test for two groups and one-way ANOVA with LSD *post-hoc* test for multiple groups, using SPSS software. Source data are provided as a Source Data file.

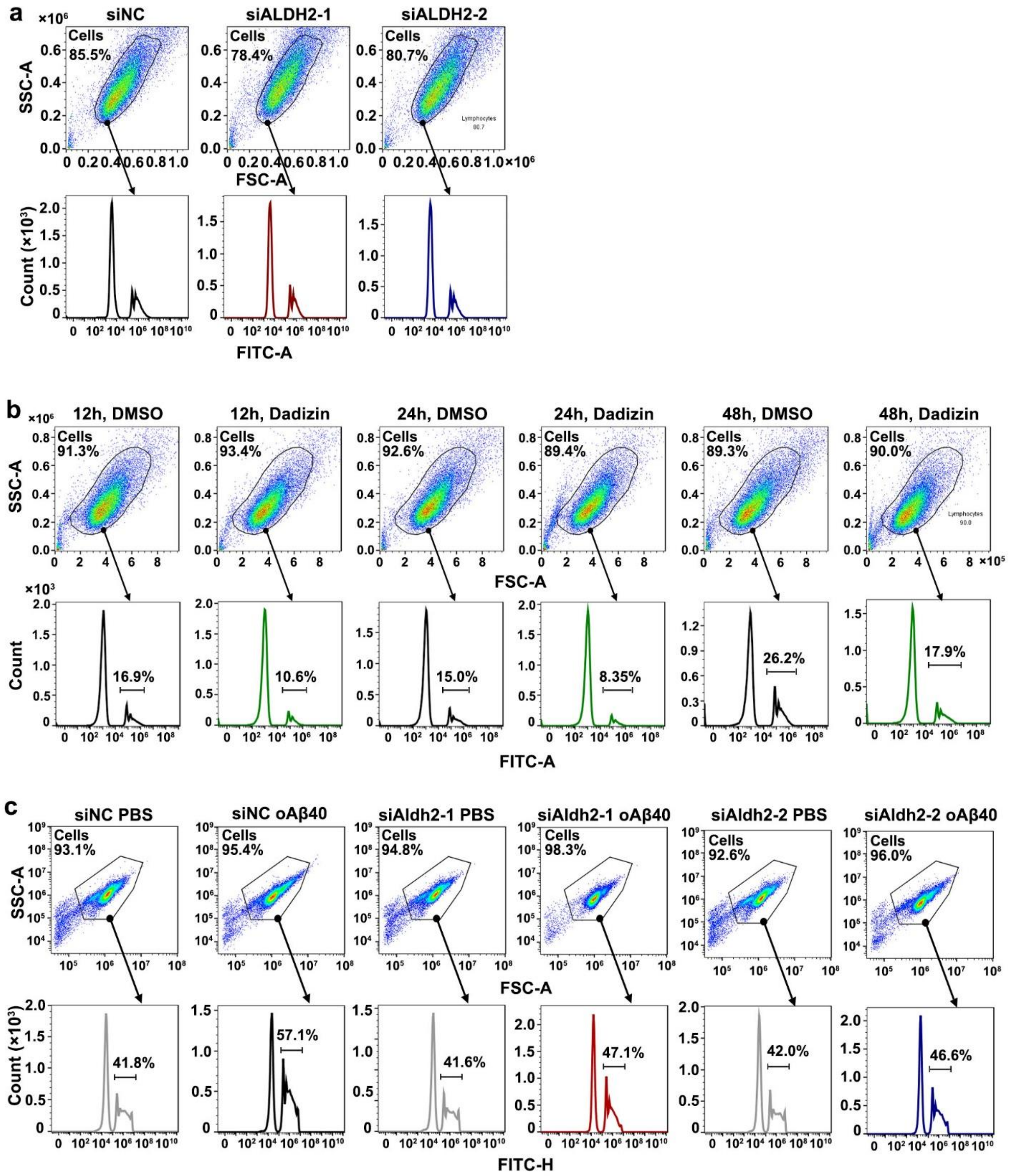

Supplementary Figure 13. Flow cytometry gating strategy.

a, Gating strategy for the phagocytosis rate of latex beads of BV2 cells with Aldh2 knockdown, associated with **Supplementary Figure 12e**. **b**, Gating strategy for the phagocytosis rate of latex beads of BV2 cells stimulated with daidzin (20 μM) for 12, 24, and 48 h, associated with **Supplementary Figure 12f**. **c**, Gating strategy for the phagocytosis rate of BV2 cells with Aldh2

knockdown, stimulated with oAβ40 (1 μM) for 24 h and then co-incubated with latex beads for 4 h associated with **Figure 7e**.

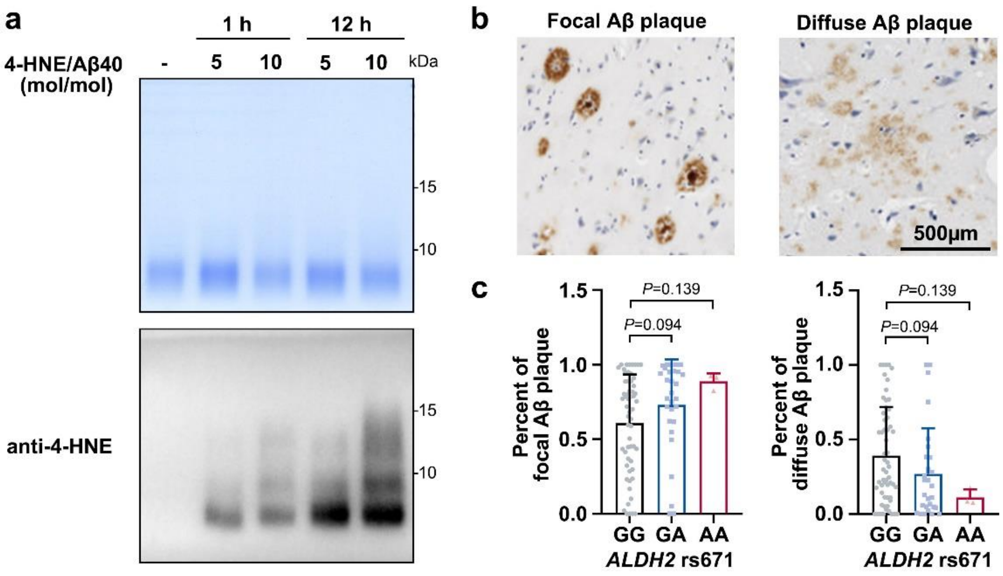

Supplementary Figure 14. 4-HNE promoted aggregation of Aβ *in vitro* and *in vivo*.

a, 4-HNE treatment enhances Aβ40 peptide oligomerization. Coomassie blue staining and WB of 4-HNE adducts with 4-HNE antibody. **b–c,** Representative images **(b)** and percentage **(c)** of focal Aβ plaques and diffuse Aβ plaques by *ALDH2* rs671 polymorphism. rs671 GG genotype (n = 58, 31M and 27F, 84.00 ± 7.90 y); GA (n = 29, 20M and 9F, 83.90 ± 8.15 y); AA (n = 3, 1M and 2F, 84.95 ± 9.35 y). Data are presented as mean values ± SD. Statistical analysis was performed using one-way ANOVA with LSD *post-hoc* test for multiple groups. Source data are provided as a Source Data file.

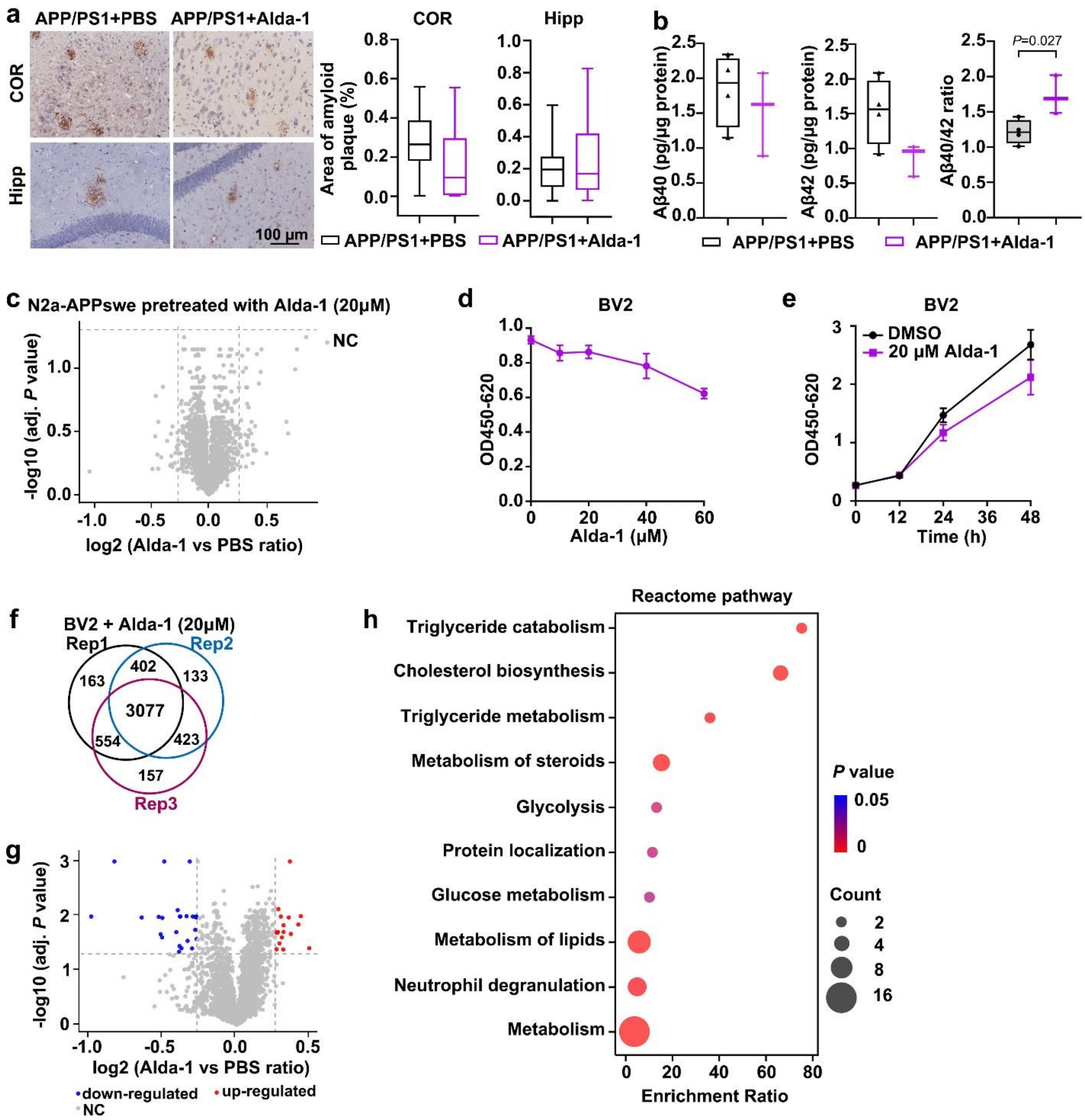

Supplementary Figure 15. Functions of ALDH2 activator Alda-1.

a–b, (a) Immunohistochemical staining of Aβ plaque deposition in the cortex (COR) and hippocampus (Hipp), and **(b)** levels of Aβ40 and Aβ42 peptides in the cortex, from APP/PS1 transgenic mice treated with Alda-1 (15 mg/kg/day) for 2 months (n = 3) and age-matched untreated APP/PS1 mice (n = 4). Scale bar, 100 μm. **c**, Volcano plot of all confident proteins identified in the proteomic data of N2a-APPswe cells pretreated with 20 μM Alda-1 for 24 h. **d–e,** Proliferation of BV2 cells treated with **(d)** 20, 40, or 60 μM Alda-1 for 6 h (n = 3–4), or **(e)** 20 μM

Alda-1 for 12, 24, 36, or 48 h (n = 7–8). **f-h**, Proteomic analysis of proteins in BV2 cells pretreated with 20 μM Alda-1 for 24h. (**f**) Venn map of confident proteins. (**g**) volcano plot. The threshold for differentially expressed proteins screening was set as: adjusted P value (BH corrected) ≥ 0.05, fold change ≤ 0.83 or ≥ 1.20. (**h**) Reactome pathway analysis of differentially expressed proteins. Data are presented as mean values ± SD. Statistical analysis was performed using two-tailed Student's *t*-test for two groups. Source data are provided as a Source Data file.

Supplementary Table 1. Relationships between *ALDH2* rs671 genotypes and sex.

	n	**Number of rs671 genotype (frequency%)**		***P***
		**GG**	**GA/AA**	
Male	267	183 (39.0)	84 (17.9)	0.219
Female	202	149 (31.8)	53 (11.3)	

P: Bivariate spearman correlation coefficient, two-tailed test of significance, using SPSS.

Supplementary Table 2. Association of risk factors with *ALDH2* rs671 polymorphism and Alzheimer's disease (AD)-related neuropathologic changes after adjustment for age in Chinese populations.

rs671	Male		Female	
	**Odds ratio (95% CI)**	***P* value**	**Odds ratio (95% CI)**	***P* value**
n	267 (183GG:79GA:5AA)		202 (149GG:44GA:9AA)	
**AD neuropathologic change**				
GG	1.00		1.00	
GA	1.09 (0.64-1.83)	0.76	1.23 (0.65-2.33)	0.52
AA	2.18 (0.41-11.68)	0.36	2.00 (0.57-7.04)	0.28
GA/AA	1.15 (0.69-1.91)	0.60	1.35 (0.74-2.43)	0.33
**Aβ plaque score (A score)**				
GG	1.00		1.00	
GA	1.11 (0.66-1.86)	0.70	1.03 (0.55-1.92)	0.92
AA	6.33 (1.12-35.63)	**0.04***	2.24 (0.66-7.60)	0.20
GA/AA	1.23 (0.74-2.03)	0.43	1.18 (0.66-2.11)	0.57
**Braak NFT stage score (B score)**				
GG	1.00		1.00	
GA	1.17 (0.68-2.02)	0.57	1.07 (0.54-2.10)	0.85
AA	0.71 (0.12-4.27)	0.71	2.85 (0.74-11.00)	0.13
GA/AA	1.13 (0.66-1.93)	0.65	1.28 (0.68-2.41)	0.44
**CERAD Neuritic plaque score (C score)**				
GG	1.00		1.00	
GA	1.12 (0.65-1.92)	0.68	1.40 (0.73-2.68)	0.31
AA	3.83 (0.70-20.97)	0.12	1.43 (0.40-5.14)	0.58
GA/AA	1.22 (0.72-2.07)	0.46	1.41 (0.77-2.57)	0.27
**Average ECog score**				
GG	1.00		1.00	
GA	1.07 (0.47-2.43)	0.88	0.54 (0.19-1.52)	0.24
AA	0.69 (0.07-6.61)	0.75	0.99 (0.12-8.44)	0.99
GA/AA	1.03 (0.46-2.30)	0.94	0.60 (0.22-1.59)	0.30

Odds ratios and *P* value were calculated by ordinal logistic regression with adjustment of age using SPSS software. *n* = 469.

Supplementary Table 3. Association of risk factors with *ALDH2* rs671 polymorphism and co-neuropathologic changes after adjustment for age in Chinese populations.

rs671	*n*	Odds ratio (95% CI)	*P* value
**Lewy bodies (*n*=447)**			
GG	315	1.00	
GA	119	1.22 (0.75-1.98)	0.41
AA	13	2.51 (0.82-7.64)	0.11
GA/AA	132	1.32 (0.83-2.09)	0.24
**Braak staging of Parkinson's disease (*n*=469)**			
GG	332	1.00	
GA	123	1.37 (0.87-2.14)	0.17
AA	14	2.03 (0.69-5.99)	0.20
GA/AA	137	1.42 (0.92-2.18)	0.11
**TDP-43 pathology (*n*=446)**			
GG	320	1.00	
GA	114	0.86 (0.53-1.39)	0.54
AA	12	1.37 (0.37-5.02)	0.64
GA/AA	126	0.90 (0.56-1.43)	0.66
**Primary age-related tauopathy (*n*=465)**			
GG	330	1.00	
GA	122	1.23 (0.78-1.92)	0.37
AA	13	3.09 (0.67-14.18)	0.15
GA/AA	135	1.32 (0.85-2.04)	0.21
**Cerebral amyloid angiopathy (*n*=380)**			
GG	274	1.00	
GA	97	0.72 (0.42-1.24)	0.23
AA	9	1.16 (0.27-4.96)	0.84
GA/AA	106	0.75 (0.45-1.26)	0.28

Odds ratios and *P* value were calculated by ordinal logistic regression with adjustment of age using SPSS software.

Supplementary Table 4. Distribution of sex and age of individuals included in this study.

	Total included			IHC/Elisa		
**rs671**	AA	GA	GG	AA	GA	GG
n	14	123	332	8	18	18
**Sex**	5M:9F	79M:44F	183M:149F	3M:5F	9M:9F	9M:9F
**Age, y**	80.36 ± 10.83	81.04 ± 13.48	77.86 ± 14.57	84.62 ± 9.76	84.78 ± 4.40	82.89 ± 7.76

Supplementary Table 5. Area of Aβ plaques (%) in postmortem human brain regions with rs671 polymorphism.

	rs671 polymorphism			***P* value**
	**GG**	**GA**	**AA**	
n (sex)	18 (9M:9F)	18 (9M:9F)	8 (3M:5F)	
**Inferior parietal lobule (IPL)**				
total	0.136	1.301	1.237	**0.008****
M	0.174	1.575	1.472	**0.046***
F	0.098	1.027	1.095	0.189
**Middle frontal gyrus (MFG)**				
total	0.140	0.979	0.692	**0.003****
M	0.178	0.808	0.820	0.188
F	0.101	1.150	0.615	**0.013***
**Superior temporal gyrus (STG)**				
total	0.105	1.130	0.831	**0.001****
M	0.116	1.354	1.383	**0.018***
F	0.093	0.931	0.500	**0.016***
**Visual cortex (VC)**				
total	0.050	0.592	0.878	**0.000*****
M	0.066	0.710	0.831	**0.001****
F	0.033	0.460	0.907	**0.002****
**Hippocampus (Hipp)**				
total	0.168	0.922	0.765	**0.001****
M	0.102	1.040	1.303	**0.014***
F	0.234	0.816	0.443	**0.009****
**Basal ganglia (BG)**				
total	0.119	0.711	0.971	**0.032***
M	0.124	0.928	1.180	0.143
F	0.114	0.466	0.846	0.202
**Midbrain (Mid)**				
total	0.011	0.003	0.025	0.320
M	0.022	0.006	0.045	0.451
F	0.000	0.000	0.013	0.216
**Cerebellum (Cblm)**				
total	0.000	0.009	0.016	0.137
M	0.000	0.007	0.028	0.177
F	0.000	0.010	0.009	0.463

Statistical analysis was performed using one-way ANOVA with LSD *post-hoc* test for multiple groups. *$P < 0.05$, **$P < 0.01$, ***$P < 0.001$.

Supplementary Table 6. Elisa assay of Aβ40/42 ratio in human brain regions with rs671 polymorphism.

	rs671 polymorphism			*P* value
	**GG**	**GA**	**AA**	
n (sex)	18 (9M:9F)	18 (9M:9F)	8 (3M:5F)	
**Inferior parietal lobule (IPL)**				
total	0.142	0.168	0.178	**0.022***
M	0.149	0.178	0.206	0.089
F	0.136	0.159	0.162	0.120
**Middle frontal gyrus (MFG)**				
total	0.141	0.208	0.204	**0.000*****
M	0.132	0.205	0.176	**0.000*****
F	0.151	0.210	0.221	**0.003****
**Hippocampus (Hipp)**				
total	0.255	0.441	0.336	**0.017***
M	0.238	0.509	0.358	0.091
F	0.272	0.374	0.324	0.172

Statistical analysis was performed using one-way ANOVA with LSD *post-hoc* test for multiple groups. *$P < 0.05$, **$P < 0.01$, ***$P < 0.001$.

Supplementary Table 7. Expression of *ALDH2* in the transcriptome of human hippocampus.

**Cases**	**CTRL (*n*=19)**	**AD (*n*=31)**
**Sex**	12M:7F	15M:16F
**Age of death, years, mean ± SD**	80.84 ± 10.36	88.23 ± 5.41
***ALDH2* rs671 genotype**		
**GG**	15	20
**GA**	4	9
**AA**	0	2

Supplementary Table 8. The siRNA sequences used in this study.

siRNA	Sequence (5'→3')	Organism
siNC	GGCUCUAGAAAAGCCUAUGCdTdT	human/mouse
siVPS35-1	GTTGTTATGTGCTTAGTAA	human
siVPS35-2	GTTGTAAACTGTAGGGATG	human
siVPS35-3	GAACATATTGCTACCAGTA	human
siALDH2-1	GAGCCAACAATTCCACGTA	human
siALDH2-2	GATGAAACTCAGTTTAAGA	human
siALDH2-3	GGAGACTTCTTCAGCTACA	human
siAldh2-1	CAGCAACCTCAAGAGAGTA	mouse
siAldh2-2	GATGAAACTCAGTTTAAGA	mouse

Supplementary Table 9. The primer sequences used in this study.

Gene	Primer	Sequence (5'→3')	Organism
Il-6	Forward	TACCACTTCACAAGTCGGAGGC	mouse
	Reverse	CTGCAAGTGCATCATCGTTGTTC	
Il-1β	Forward	TGGACCTTCCAGGATGAGGACA	mouse
	Reverse	GTTCATCTCGGAGCCTGTAGTG	
Tnf-α	Forward	GGTGCCTATGTCTCAGCCTCTT	mouse
	Reverse	GCCATAGAACTGATGAGAGGGAG	
Aldh2	Forward	GCTGTTGTACCGATTGGCGGAT	mouse
	Reverse	GCGGAGACATTTCAGGACCATG	
App	Forward	TCCGTGTGATCTACGAGCGCAT	mouse
	Reverse	GCCAAGACATCGTCGGAGTAGT	
Ps1	Forward	GAGACTGGAACACAACCATAGCC	mouse
	Reverse	AGAACACGAGCCCGAAGGTGAT	
Gapdh	Forward	CATCACTGCCACCCAGAAGACTG	mouse
	Reverse	ATGCCAGTGAGCTTCCCGTTCAG	
VPS35	Forward	TGCTGATGAGCAGAGCCTTGTG	human
	Reverse	CAGTGTGAAGCGAATCCGCTGA	
ALDH2	Forward	TTGCCTCCCATGAGGATGTGGA	human
	Reverse	GGTCACTCTCTTGAGGTTGCTG	
APP	Forward	CCTTCTCGTTCCTGACAAGTGC	human
	Reverse	GGCAGCAACATGCCGTAGTCAT	
PS1	Forward	GCAGTATCCTCGCTGGTGAAGA	human
	Reverse	CAGGCTATGGTTGTGTTCCAGTC	
ACTB	Forward	CACCATTGGCAATGAGCGGTTC	human
	Reverse	AGGTCTTTGCGGATGTCCACGT	

Supplementary Table 10. Minimum recommended brain regions to be sampled and evaluated[1].

Region	AD Neuropathologic Change		
	**A**<br>**Stain for Aβ/amyloid plaques**	**B**<br>**Stain for NFTs**	**C**<br>**Stain for NPs**
Midbrain including SN (Mid)	3°: if 2° is +		
Cerebellar cortex (Cblm)	3°: if 2° is +		
Basal ganglia at level of AC with basal nucleus of Meynert (BG)	2°: if 1° is +	Consider	
Hippocampus and EC (Hipp)	2°: if 1° is +	Yes	Consider
Middle frontal gyrus (MFG)	1°	Yes	Yes
Superior and middle temporal gyri (STG)	1°	Yes	Yes
Inferior parietal lobule (IPL)	1°	Yes	Yes
Occipital cortex (BA 17 and 18), that is visual cortex (VC)	Consider	Yes	Consider

Supplementary Table 11. "ABC" score for level of AD neuropathologic change.

**AD Neuropathologic Change**		**B**		
**A**	**C**	0 or 1	2	3
0	0	Not	Not	Not
1	0 or 1	Low	Low	Low
	2 or 3	Low	Intermediate	Intermediate
2	Any C	Low	Intermediate	Intermediate
3	0 or 1	Low	Intermediate	Intermediate
	2 or 3	Low	Intermediate	High

Supplementary Table 12. ***ALDH2*** **rs671 polymorphism.**

**Gene name**	***ALDH2*, Aldehyde dehydrogenase 2 family member**		
**Accession number**		NM_000690.4	
		rs671 variant	
**Nucleotide/Allele change**	G	A	
**Amino acid**	Glu504	Lys504	
**Genotype**	GG	GA	AA
**Enzyme activity**	100%	<50%	<1-4%

Supplementary Table 13. Nonstandard Abbreviations and Acronyms.

**Abbreviations**	**Full name**
4-HNE	4-Hydroxy-2-noneal
AD	Alzheimer's disease
ALDH2	Mitochondrial aldehyde dehydrogenase 2
Annexin V-FITC	Fluorescein isothiocyanate-conjugated Annexin V
APP	Amyloid-beta precursor protein
Aβ	Amyloid beta
BG	Basal ganglia
BSA	Bovine serum albumin
Cblm	Cerebellum
CCK8	Cell Counting Kit-8
CI	Confidence interval
COR	Cortex
Cryo-EM	Cryo-electron microscopy
DEPs	Differentially expressed proteins
DMEM	Dulbecco's Modified Eagle's Medium
Ecog	Everyday cognition
ELISA	Enzyme-linked immunosorbent assay
ER	Endoplasmic reticulum
fAβ40	Fibrillar Aβ40
GSH	Glutathione
GST	Glutathione S-transferase
Hipp	Hippocampus
HNE-substrate	(*R*)-4-HNE modified substrate from N2a-APP pretreated with (*R*)-4-HNE
HPLC	High-performance liquid chromatography
IOD	Integrated optical density
IPL	Inferior parietal lobule
LPS	Lipopolysaccharide

MALDI-TOF MS	Matrix assisted laser desorption/ionization-time of flight mass spectrometry
MFG	Middle frontal gyrus
Mid	Midbrain
NIA/AA	National Institute on Aging/Alzheimer Association
oAβ40	Oligomeric Aβ40
OR	Odds ratio
PBS	Phosphate-buffered saline
PFA	Paraformaldehyde
PI	Propidium iodide
RT-qPCR	Quantitative real-time PCR
STG	Superior temporal gyrus
TGN	Trans-Golgi network
TM	Transmembrane
VC	Visual cortex
WB	Western blot
WT	Wild-type
WT-substrate	Wild-type substrate from N2a-APP

Supplementary Methods

Brain tissue preparation

Following the complete extraction of the whole brain from the cranial cavity, an assessment of its weight, volume, and overall morphology was conducted, capturing the characteristics of the cerebral hemispheres. Subsequently to the removal of the dura mater and major cerebral blood vessels, the whole brain, including the cerebrum, cerebellum, and brainstem, was carefully divided along the mid-sagittal plane, establishing distinct left and right hemispheres. The left hemisphere was subsequently subjected to the freezing procedure, while the right hemisphere underwent formalin fixation. It is noteworthy that these meticulous steps were executed in strict accordance with the "*Standardized Operational Protocol for Human Brain Banking in China*"[2].

Neuropathological evaluation

According to the guidelines of the National Institute on Aging/Alzheimer Association[1], all brain tissues received identical neuropathological analysis by the "ABC" score (**Supplementary Table 10**). The A score reflects the brain regions extend of Aβ appearance in the brain. B score represents the NFT stage, C score is the neuritic plaque score. AD neuropathologic change is evaluated by the combination of A, B, and C scores and is designated as "Not/N", "Low/L", "Intermediate/I" or "High/H". A neuropathological AD score of N/L indicates that the donor is unlikely to have AD and can be regarded as a normal elderly person. A neuropathological AD score of I/H indicates that the donor is very likely to have AD (**Supplementary Table 11**).

Lewy bodies. The assessment of probable dementia with Lewy bodies was based on the 2005 McKeith criteria[3]. Five stages (N/B/T/D/A) were used to assess the regional pattern of Lewy-

related pathology. N, none; B, Brainstem-predominant; T, Limbic (transitional); D, Diffuse neocortical; A, Amygdala predominant.

Braak staging of Parkinson's disease. The Braak staging (α-synuclein) was used to assess the neuropathology of Parkinson's disease according to Braak (2003) [4]. Seven stages (0-7) were used to evolute the regional pattern of PD-related pathology.

TDP-43 pathology. TDP-43 pathology (score 0-3) was assessed by TDP-43 immunohistochemical staining of amygdala, hippocampus, and middle frontal gyrus, according to Nelson (2019)[5].

Primary age-related tauopathy. Primary age-related tauopathy was assessed according to Crary (2014)[6].

Cerebral amyloid angiopathy. Cerebral amyloid angiopathy is characterized by the deposition of the amyloid β-protein (Aβ) within cerebral vessels, and is assessed according to Thal (2008)[7].

Cognitive function assessment

Clinical cognitive status was determined using the Everyday Cognitive (ECog) Insider Questionnaire, which includes 39 questions aimed to assess the daily cognitive function of the brain donors. In accordance with the criteria for ECog scores, normal cognition was defined as an ECog score ≤1.0, mild cognitive impairment as an ECog score 1.0–2.0, and dementia as an ECog score ≥2.0[8].

Supplementary Notes. Glossary.

***ALDH2* rs671 polymorphism**

Human Aldehyde dehydrogenase 2 family member (ALDH2) is a 517-amino acid polypeptide encoded by a nuclear gene located at chromosome 12q24. The protein is transported to the mitochondrial matrix in a process that is dependent on its *N* terminus 17-amino acid mitochondrial targeting sequence, which is cleaved as part of the complete folding and maturation of the enzyme inside the mitochondria.

It is reported that 535 coding single-nucleotide polymorphisms have been identified in the *ALDH2* gene. The rs671 variant is caused by a single G to A nucleotide change (NM_000690.4:c.1510G>A), which leads to a substitution of Glu to Lys at position 504 (E504K) in the ALDH2 protein monomer (NP_000681.2:p.Glu504Lys). This mutation dramatically decreases ALDH2 enzymatic activity, with <50% of the wild-type activity for the GA genotype, and <1-4% for the AA genotype (**Supplementary Table 12**) [9,10]. The decreased ALDH2 activity results in decreased metabolism of aldehydes, including ethanol-derived acetaldehyde, and endogenous 4-hydroxy-2-nonenal (4-HNE) and malondialdehyde.

γ-Secretase protease

γ-Secretase proteases are multimeric intramembrane proteases, with presenilin (PS1 or PS2), nicastrin (NCSTN), presenilin enhancer 2 (PEN-2) and anterior pharynx defective 1 (APH1) as essential components[11,12]. Presenilin is the catalytic subunit and contains nine transmembrane segments[13].

Aβ40 and Aβ42 generation

The amyloid precursor protein (APP) is first cleaved by β-secretase to produce a 99-residue transmembrane fragment C99. The C99 is further proteolyzed by γ-secretase complex to generate intracellular domain (AICD) and 48- or 49-residue transmembrane peptide (Aβ48 or Aβ49)[14]. Aβ48 or Aβ49 are then trimmed every three or four residues by γ-secretase, generating Aβ peptides of varying lengths (Aβ49→Aβ46→Aβ43→Aβ40→Aβ37; Aβ48→Aβ45→Aβ42→Aβ38) and byproducts of diverse tri- and tetra-peptides. Aβ40 and Aβ42 are the main components of extracellular plaques[15,16].

APP/PS1 transgenic mice

APP/PS1 (APPswePSEN1 dE9) mice contain human transgenes for both APPswe, APP bearing the so-called "Swedish mutation" (K670N/M671L) which causes early onset familial Alzheimer's disease, and human presenilin protein 1 (PS1) carrying the exon-9-deleted variant (PS1-dE9) both under the control of the Thy1 promoter. In these mice, expression of the human APP transgene is approximately threefold higher than that of endogenous murine APP. The plaque deposition starts at about six weeks of age in the cortex and three to four months of age in the hippocampus. The model also shows phosphorylated tau-positive neurites around the plaques, but no fibrillar tau tangles. The APP/PS1 mice exhibit cognitive impairments in spatial learning and memory tasks, such as the Morris water maze and the four-arm spatial maze. They also show impairments in long-term potentiation (LTP) in the hippocampus and modest neuron loss in some brain regions at older ages. The APP/PS1 mouse model is widely used to study the mechanisms of amyloid pathology and to test potential therapeutic interventions for Alzheimer's disease[17].

Supplementary References

1 Montine, T. J. et al. National Institute on Aging-Alzheimer's Association guidelines for the neuropathologic assessment of Alzheimer's disease: a practical approach. *Acta Neuropathol.* **123**, 1-11 (2012).

2 Qiu, W. et al. Standardized operational protocol for human brain banking in China. *Neurosci. Bull.* **35**, 270-276 (2019).

3 McKeith, I. G. et al. Diagnosis and management of dementia with Lewy bodies: third report of the DLB Consortium. *Neurology* **65**, 1863-1872 (2005).

4 Braak, H. et al. Staging of brain pathology related to sporadic Parkinson's disease. *Neurobiol. Aging* **24**, 197-211 (2003).

5 Nelson, P. T. et al. Limbic-predominant age-related TDP-43 encephalopathy (LATE): consensus working group report. *Brain* **142**, 1503-1527 (2019).

6 Crary, J. F. et al. Primary age-related tauopathy (PART): a common pathology associated with human aging. *Acta Neuropathol.* **128**, 755-766 (2014).

7 Thal, D. R., Griffin, W. S., de Vos, R. A. & Ghebremedhin, E. Cerebral amyloid angiopathy and its relationship to Alzheimer's disease. *Acta Neuropathol.* **115**, 599-609 (2008).

8 Yang, Q. et al. Correlations between single nucleotide polymorphisms, cognitive dysfunction, and postmortem brain pathology in Alzheimer’s disease among Han Chinese. *Neurosci. Bull.* **35**, 193-204 (2019).

9 Zhang, J. et al. The role of aldehyde dehydrogenase 2 in cardiovascular disease. *Nat. Rev. Cardiol.* **20**, 495-509 (2023).

10 Chen, C. H., Ferreira, J. C., Gross, E. R. & Mochly-Rosen, D. Targeting aldehyde dehydrogenase 2: new therapeutic opportunities. *Physiol. Rev.* **94**, 1-34 (2014).

11 Sun, L. et al. Structural basis of human gamma-secretase assembly. *Proc. Natl. Acad. Sci. USA* **112**, 6003-6008 (2015).

12 Lu, P. et al. Three-dimensional structure of human gamma-secretase. *Nature* **512**, 166-170, doi:10.1038/nature13567 (2014).

13 Petit, D. *et al.* Aβ profiles generated by Alzheimer's disease causing PSEN1 variants determine the pathogenicity of the mutation and predict age at disease onset. *Mol Psychiatry* **27**, 2821-2832 (2022).

14 Yang, G. et al. Structural basis of Notch recognition by human gamma-secretase. *Nature* **565**, 192-197 (2019).

15 Zhou, R. et al. Recognition of the amyloid precursor protein by human γ-secretase. *Science* ***363,*** eaaw0930 (2019).

16 Takami, M. et al. gamma-Secretase: successive tripeptide and tetrapeptide release from the transmembrane domain of beta-carboxyl terminal fragment. *J. Neurosci.* **29**, 13042-13052 (2009).

17 Lok, K. et al. Characterization of the APP/PS1 mouse model of Alzheimer's disease in senescence accelerated background. *Neurosci. Lett.* **557 Pt B**, 84-89 (2013).
