# Supplementary material for: The aldehyde dehydrogenase 2 rs671 variant enhances amyloid β pathology
Source: Nat Commun. 2024 Mar 22;15:2594. doi: 10.1038/s41467-024-46899-0 (PMC10959958; doi:10.1038/s41467-024-46899-0)
Supplement: Supplementary file 3 — Description of Additional Supplementary Files [file 41467_2024_46899_MOESM3_ESM.pdf]

Description of Additional Supplementary Files:

Supplementary Data 1: Human brain samples used in the experiments in this study.

Supplementary Data 2: Proteomics data of N2a-APPswe cells with treatment of daidzin or Alda-1.

Supplementary Data 3: Proteomics data of BV2 cells with treatment of Alda-1.
