# Supplementary material for: The aldehyde dehydrogenase 2 rs671 variant enhances amyloid β pathology
Source: Nat Commun. 2024 Mar 22;15:2594. doi: 10.1038/s41467-024-46899-0 (PMC10959958; doi:10.1038/s41467-024-46899-0)
Supplement: Supplementary file 7 — Reporting Summary [file 41467_2024_46899_MOESM7_ESM.pdf]

nature portfolio

Corresponding author(s): Wei Ge

Last updated by author(s): Feb 19, 2024

Reporting Summary

Nature Portfolio wishes to improve the reproducibility of the work that we publish. This form provides structure for consistency and transparency in reporting. For further information on Nature Portfolio policies, see our Editorial Policies and the Editorial Policy Checklist.

Statistics

For all statistical analyses, confirm that the following items are present in the figure legend, table legend, main text, or Methods section.

n/a	Confirmed	
☐	☒	The exact sample size (*n*) for each experimental group/condition, given as a discrete number and unit of measurement
☐	☒	A statement on whether measurements were taken from distinct samples or whether the same sample was measured repeatedly
☐	☒	The statistical test(s) used AND whether they are one- or two-sided<br>*Only common tests should be described solely by name; describe more complex techniques in the Methods section.*
☒	☐	A description of all covariates tested
☒	☐	A description of any assumptions or corrections, such as tests of normality and adjustment for multiple comparisons
☐	☒	A full description of the statistical parameters including central tendency (e.g. means) or other basic estimates (e.g. regression coefficient) AND variation (e.g. standard deviation) or associated estimates of uncertainty (e.g. confidence intervals)
☐	☒	For null hypothesis testing, the test statistic (e.g. *F*, *t*, *r*) with confidence intervals, effect sizes, degrees of freedom and *P* value noted<br>*Give P values as exact values whenever suitable.*
☒	☐	For Bayesian analysis, information on the choice of priors and Markov chain Monte Carlo settings
☒	☐	For hierarchical and complex designs, identification of the appropriate level for tests and full reporting of outcomes
☒	☐	Estimates of effect sizes (e.g. Cohen's *d*, Pearson's *r*), indicating how they were calculated

Our web collection on statistics for biologists contains articles on many of the points above.

Software and code

Policy information about availability of computer code

Data collection	Mass spectrometry (MS)-based proteomics data were acquired on an Orbitrap Fusion Tribrid Mass spectrometer (Thermo Fisher Scientific, USA, Xcalibur software, v2.1.2). Immunofluorescent images were captured by Leica TCS SP8 STED 3X (Leica Microsystems, inc, Leica Application Suite X v3.7.3.23245) and DMi8 Inverted microscope (Leica DMi8; Leica Microsystems, inc, Leica Application Suite X v3.7.3.23245). MOLDI-TOF was performed on 4800 Plus MALDI TOF/TOFTM Analyzer (AB SCIEX, USA). Flow cytometry assay was performed on Flow cytometer Cytoflex (Beckman, USA, CytExpert software, v2.4.028). qPCR data were acquired on a CFX ConnectTM Real Time System (Bio-Rad CFX Maestro 2.2 v5.2.008.0222, Bio-Rad Laboratories). The structure of proteins were downloaded from AlphaFold Protein Structure Database (https://alphafold.ebi.ac.uk/). Westernblot images were captured by ChemiDoc XRS+ System (Bio-Rad Image Lab software, Version 6.1.0 Build 7, Bio-Rad Laboratories). ELISA signals were measured on a Multiskan™ FC Microplate Photometer (Thermo Fisher Scientific).
Data analysis	Proteomics data were analysed by Proteome Discoverer software (Thermo Fisher Scientific, USA, Version 2.4.1.15), JMP Pro software (SAS Institute, USA, Version 13.2.1) and Gephi (Version 0.10.1). Image-Pro Plus software (Media Cybernetics, USA, Version 6.0) was used to analyze immunofluorescence and IHC images. Data of the flow cytometry assay were analysed with FlowJo analytical software (Becton, Dickinson & Company, USA, vX.0.7). Protein-protein docking was performed by ZDOCK software (Version 3.0.2) and Rosetta (Version 2021.16). Sequence alignment was done in PyMOL (Version 2.5.2). Statistical analyses were performed with the GraphPad Prism 8.4.3 (686) and IBM® SPSS® statistics software (IBM, USA, Version 25.0).

For manuscripts utilizing custom algorithms or software that are central to the research but not yet described in published literature, software must be made available to editors and reviewers. We strongly encourage code deposition in a community repository (e.g. GitHub). See the Nature Portfolio guidelines for submitting code & software for further information.

Data

Policy information about availability of data

All manuscripts must include a data availability statement. This statement should provide the following information, where applicable:

- Accession codes, unique identifiers, or web links for publicly available datasets
- A description of any restrictions on data availability
- For clinical datasets or third party data, please ensure that the statement adheres to our policy

Data Availability
The mass spectrometry proteomics data generated in this study have been deposited to the ProteomeXchange Consortium via the iProX partner repository67,68 with the dataset identifier PXD047209 [https://proteomecentral.proteomexchange.org/cgi/GetDataset?ID=PXD047209] and PXD047210 [https://proteomecentral.proteomexchange.org/cgi/GetDataset?ID=PXD047210].
The raw sequence data reported in this paper have been deposited in the Genome Sequence Archive (Genomics, Proteomics & Bioinformatics 2021) in National Genomics Data Center (Nucleic Acids Res 2022), China National Center for Bioinformation/Beijing Institute of Genomics, Chinese Academy of Sciences, the accession number HRA006714 [https://ngdc.cncb.ac.cn/gsa-human]), under controlled access. Data are available from the corresponding author upon request and with no delay for data sharing.
The published structure of free C99 (PDB: 2LLM[https://doi.org/10.2210/pdb2LLM/pdb]) and the structure of C99 bound to γ-secretase (PDB: 6IYC [https://doi.org/10.2210/pdb6IYC/pdb]) were used in Fig 4e.
Source data are available on Figshare (https://doi.org/10.6084/m9.figshare.24647757) and provided with the article.

Research involving human participants, their data, or biological material

Policy information about studies with human participants or human data. See also policy information about sex, gender (identity/presentation), and sexual orientation and race, ethnicity and racism.

Reporting on sex and gender	Brain tissues from 267 males and 202 females (a total of 469 samples) were used in this study. 44 brain tissues (21 males and 23 females) were used for IHC and Elisa assay. Detailed information was included in Supplementary Data 1. Findings did not apply to only one sex.
Reporting on race, ethnicity, or other socially relevant groupings	This study enrolled only Chinese population cohort due to the significant regional preference (30-50%) of ALDH2 rs671 G>A mutation in the East Asian populations, while it was less than 5% of population in other countries.
Population characteristics	Postmortem brains from donors with sanger sequencing of ALDH2 rs671 polymorphisms (GG, GA, or AA genotype), and with "ABC" pathology scores were used in this study. Please see Supplementary Data 1 for detailed information.
Recruitment	No Living Human research has been performed. Therefore, this is N/A.
Ethics oversight	All samples were donated with the full, informed consent. The use of post mortem brains in this study was approved by the Ethics Committee of the Institute of Basic Medical Sciences Chinese Academy of Medical Sciences

Note that full information on the approval of the study protocol must also be provided in the manuscript.

Field-specific reporting

Please select the one below that is the best fit for your research. If you are not sure, read the appropriate sections before making your selection.

☒ Life sciences ☐ Behavioural & social sciences ☐ Ecological, evolutionary & environmental sciences

For a reference copy of the document with all sections, see nature.com/documents/nr-reporting-summary-flat.pdf

Life sciences study design

All studies must disclose on these points even when the disclosure is negative.

Sample size	No statistical methods were used to predetermine sample size. Sample sizes were similar to those reported in previous publications (Cameron et al, Natue, 2022; Dan, et al, Aging Dis, 2023). For animals or postmortem brains study, sample size "n" represents the number of animals or brain samples used. For in vitro study, sample size "n" represents the number of independent samples.
Data exclusions	No data were excluded for analysis.
Replication	All experiments were replicated in at least three independent experiments unless stated otherwise. The exact number of repetitions (individual data points from each cell and/or tissue) are indicated in figures or figure legends.
Randomization	For in vivo experiments, animals were randomly assigned for drug treatment to cover sex/drugs/etc for balancing potential batch effects. The allocation of postmortem brains was random in each genotype. Cell culture experiments with cell lines were handled the same way.

Blinding	The investigators were not blinded to all conditions as they were responsible for both experimental design and data collection. The samples were not blinded during initial study planning to ensure that the number of groups of mice were randomized and balanced while age and sex matched. Image collection and analysis on postmortem brains, mouse brains and cell lines were performed by another author in a blinded fashion. Other experiments including PCR and FACS analysis, the collectors were blind from group information to samples.

Behavioural & social sciences study design

All studies must disclose on these points even when the disclosure is negative.

Study description	*Briefly describe the study type including whether data are quantitative, qualitative, or mixed-methods (e.g. qualitative cross-sectional, quantitative experimental, mixed-methods case study).*
Research sample	*State the research sample (e.g. Harvard university undergraduates, villagers in rural India) and provide relevant demographic information (e.g. age, sex) and indicate whether the sample is representative. Provide a rationale for the study sample chosen. For studies involving existing datasets, please describe the dataset and source.*
Sampling strategy	*Describe the sampling procedure (e.g. random, snowball, stratified, convenience). Describe the statistical methods that were used to predetermine sample size OR if no sample-size calculation was performed, describe how sample sizes were chosen and provide a rationale for why these sample sizes are sufficient. For qualitative data, please indicate whether data saturation was considered, and what criteria were used to decide that no further sampling was needed.*
Data collection	*Provide details about the data collection procedure, including the instruments or devices used to record the data (e.g. pen and paper, computer, eye tracker, video or audio equipment) whether anyone was present besides the participant(s) and the researcher, and whether the researcher was blind to experimental condition and/or the study hypothesis during data collection.*
Timing	*Indicate the start and stop dates of data collection. If there is a gap between collection periods, state the dates for each sample cohort.*
Data exclusions	*If no data were excluded from the analyses, state so OR if data were excluded, provide the exact number of exclusions and the rationale behind them, indicating whether exclusion criteria were pre-established.*
Non-participation	*State how many participants dropped out/declined participation and the reason(s) given OR provide response rate OR state that no participants dropped out/declined participation.*
Randomization	*If participants were not allocated into experimental groups, state so OR describe how participants were allocated to groups, and if allocation was not random, describe how covariates were controlled.*

Ecological, evolutionary & environmental sciences study design

All studies must disclose on these points even when the disclosure is negative.

Study description	*Briefly describe the study. For quantitative data include treatment factors and interactions, design structure (e.g. factorial, nested, hierarchical), nature and number of experimental units and replicates.*
Research sample	*Describe the research sample (e.g. a group of tagged Passer domesticus, all Stenocereus thurberi within Organ Pipe Cactus National Monument), and provide a rationale for the sample choice. When relevant, describe the organism taxa, source, sex, age range and any manipulations. State what population the sample is meant to represent when applicable. For studies involving existing datasets, describe the data and its source.*
Sampling strategy	*Note the sampling procedure. Describe the statistical methods that were used to predetermine sample size OR if no sample-size calculation was performed, describe how sample sizes were chosen and provide a rationale for why these sample sizes are sufficient.*
Data collection	*Describe the data collection procedure, including who recorded the data and how.*
Timing and spatial scale	*Indicate the start and stop dates of data collection, noting the frequency and periodicity of sampling and providing a rationale for these choices. If there is a gap between collection periods, state the dates for each sample cohort. Specify the spatial scale from which the data are taken*
Data exclusions	*If no data were excluded from the analyses, state so OR if data were excluded, describe the exclusions and the rationale behind them, indicating whether exclusion criteria were pre-established.*
Reproducibility	*Describe the measures taken to verify the reproducibility of experimental findings. For each experiment, note whether any attempts to repeat the experiment failed OR state that all attempts to repeat the experiment were successful.*
Randomization	*Describe how samples/organisms/participants were allocated into groups. If allocation was not random, describe how covariates were controlled. If this is not relevant to your study, explain why.*
Blinding	*Describe the extent of blinding used during data acquisition and analysis. If blinding was not possible, describe why OR explain why blinding was not relevant to your study.*

Did the study involve field work? ☐ Yes ☐ No

Field work, collection and transport

Field conditions	*Describe the study conditions for field work, providing relevant parameters (e.g. temperature, rainfall).*
Location	*State the location of the sampling or experiment, providing relevant parameters (e.g. latitude and longitude, elevation, water depth).*
Access & import/export	*Describe the efforts you have made to access habitats and to collect and import/export your samples in a responsible manner and in compliance with local, national and international laws, noting any permits that were obtained (give the name of the issuing authority, the date of issue, and any identifying information).*
Disturbance	*Describe any disturbance caused by the study and how it was minimized.*

Reporting for specific materials, systems and methods

We require information from authors about some types of materials, experimental systems and methods used in many studies. Here, indicate whether each material, system or method listed is relevant to your study. If you are not sure if a list item applies to your research, read the appropriate section before selecting a response.

Materials & experimental systems

n/a	Involved in the study	
☐	☒	Antibodies
☐	☒	Eukaryotic cell lines
☒	☐	Palaeontology and archaeology
☐	☒	Animals and other organisms
☒	☐	Clinical data
☒	☐	Dual use research of concern
☒	☐	Plants

Methods

n/a	Involved in the study	
☒	☐	ChIP-seq
☐	☒	Flow cytometry
☒	☐	MRI-based neuroimaging

Antibodies

Antibodies used

Westernblot：Anti-ALDH2 (Proteintech, 15310-1-AP, Polyclonal Antibody, 1:4000), anti-β-actin (GeneTex, GTX124213, Polyclonal Antibody, 1:10000), anti-GAPDH (MBL, M171-3, 3H12, 1:10000), anti-presenilin 1 (Sigma, MAB5232, clone PS1-loop, 1:500), anti-β-amyloid (1–16) (Biolegend, 803001, 6E10, 1:1000), anti-4-HNE (Abcam, ab46545, Polyclonal Antibody, 1:200), anti-LC3A (Cell Signaling Technology, 4599, D50G8, 1:1000), anti-APP C-Terminal Fragment (Biolegend, 802801, C1/6.1, 1:500), anti-APH1 (Invitrogen, PA1-2010, Polyclonal Antibody, 1:1000), anti-PEN2 (Abcam, ab18189, Polyclonal Antibody, 1:500), anti-GM130 (Cell Signaling Technology, 12480S, D6B1, 1:1000), anti-VPS35 (Abcam, ab10099, Polyclonal Antibody, 1:1000), anti-β-amyloid (1–40) (Cell Signaling Technology, 12990, D8Q7I, 1:1000), anti-β-amyloid (1–42) (Cell Signaling Technology, 14974, D9A3A, 1:1000), anti-TGN46 (Invitrogen, MA3-063, 2F7.1, 1:1000), anti-VDAC (Cell Signaling Technology, 4661S, D73D12, 1:1000), anti-Rab5 (Cell Signaling Technology, 3547, C8B1, 1:1000), anti-LAMP2 (Proteintech, 27823-1-AP, Polyclonal Antibody, 1:1000), anti-RCAS1 (Cell Signaling Technology, 12290S, D2B6N, 1:1000), anti-CANX (Cell Signaling Technology, 2679, C5C9, 1:1000), anti-tau (Cell Signaling Technology, 46687, D1M9X, 1:1000), anti-phospho-S396-tau (Abcam, ab32057, E178, 1:1000), anti-α-tubulin (GeneTex, GTX628802, GT114, 1:10000), anti-SorLA/SORL1 antibody (Abcam, ab190684, EPR14670, 1:1000), goat anti-rabbit HRP-conjugated secondary antibody (ZSGB-BIO, ZB-2301, Polyclonal Antibody, 1:10000), rabbit anti-mouse HRP-conjugated secondary antibody (ZSGB-BIO, ZB-2305, Polyclonal Antibody, 1:10000) and rabbit anti-goat HRP-conjugated secondary antibody (ZSGB-BIO, ZB-2306, Polyclonal Antibody, 1:10000)

Immunofluorescence：anti-β-amyloid (1–16) (Biolegend, 803001, 6E10, 1:200 for human brain, 1:500 for mouse brain), anti-4-HNE (Abcam, ab46545, Polyclonal Antibody, 1:200), anti-APP C-Terminal Fragment (Biolegend, 802801, C1/6.1, 1:200), anti-RCAS1 (Cell Signaling Technology, 12990S, D2B6N, 1:100), anti-Rab5 (Cell Signaling Technology, 3547, C8B1, 1:150), anti-LAMP2 (Proteintech, 27823-1-AP, Polyclonal Antibody, 1:100), anti-CANX (Cell Signaling Technology, 2679, C5C9, 1:100), anti-Iba1 (Wako, 019-19741, Polyclonal Antibody, 1:200 for human brain, 1:500 for mouse brain), Alexa FluorTM 488 goat anti-rabbit IgG (Invitrogen, A-11034, Polyclonal Antibody, 1:200), Alexa FluorTM 594 goat anti-rabbit IgG (Invitrogen, A-11037, Polyclonal Antibody, 1:200), Alexa FluorTM Plus 488 goat anti-mouse IgG (Invitrogen, A-32723, Polyclonal Antibody, 1:200), and Alexa FluorTM 594 goat anti-mouse IgG (Invitrogen, A-11032, Polyclonal Antibody, 1:200). Cell nuclei were stained using 4',6-diamidino-2-phenylindole (DAPI, Solarbio, C0065)

Coimmunoprecipitation (Co-IP):
anti-4-HNE (Abcam, ab46545, Polyclonal Antibody, 2 μg antibody in 1 ml reaction volume), anti-β-amyloid (1–16) (Biolegend, 803001, 6E10, 2 μg antibody in 1 ml reaction volume), anti-SorLA/SORL1 antibody (Abcam, ab190684, EPR14670, 2 μg antibody in 1 ml reaction volume)

Immunohistochemistry：anti-β-amyloid (1–16) (Biolegend, 803001, 6E10, 1:200), anti-4-HNE (Abcam, ab46545, Polyclonal Antibody, 1:2000), poly horseradish peroxidase anti-rabbit secondary antibody (ZSGB-BIO, PV-9001), and poly horseradish peroxidase anti-mouse secondary antibody (ZSGB-BIO, PV-9002)

Flow cytometry analysis
FITC Annexin V Apoptosis Detection Kit with PI (Biolegend, 640914)

Validation

Validation for antibodies by the company and/or studies cited on the company's websites.
Anti-ALDH2 (Proteintech, 15310-1-AP, Polyclonal Antibody):
https://www.ptglab.com/products/ALDH2-Antibody-15310-1-AP.htm
anti-β-actin (GeneTex, GTX124213, Polyclonal Antibody):
https://www.genetex.cn/PDF/Download?catno=GTX124213
anti-GAPDH (MBL, M171-3, 3H12):
https://ruo.mbl.co.jp/bio/dtl/A/?pcd=M171-3
anti-presenilin 1 (Sigma, MAB5232, clone PS1-loop):
https://www.sigmaaldrich.cn/CN/en/product/mm/mab5232
anti-β-amyloid (1–16) (Biolegend, 803001, 6E10):
https://www.biolegend.com/fr-ch/products/purified-anti-beta-amyloid-1-16-antibody-11228
anti-4-HNE (Abcam, ab46545, Polyclonal Antibody):
https://www.abcam.com/products/primary-antibodies/4-hydroxynonenal-antibody-ab46545.html
anti-LC3A (Cell Signaling Technology, 4599, D50G8, 1:1000):
https://www.cellsignal.cn/products/primary-antibodies/lc3a-d50g8-xp-rabbit-mab/4599
anti-APP C-Terminal Fragment (Biolegend, 802801, C1/6.1):
https://www.biolegend.com/en-us/clone-search/purified-anti-app-c-terminal-fragment-antibody-11275?GroupID=BLG15648
anti-APH1 (Invitrogen, PA1-2010, Polyclonal Antibody):
https://www.thermofisher.cn/cn/zh/antibody/product/APH1-Antibody-Polyclonal/PA1-2010
anti-PEN2 (Abcam, ab18189, Polyclonal Antibody):
https://www.abcam.com/products/primary-antibodies/pen2-antibody-ab18189.html
anti-GM130 (Cell Signaling Technology, 12480S, D6B1):
https://www.cellsignal.cn/products/primary-antibodies/gm130-d6b1-xp-rabbit-mab/12480
anti-VPS35 (Abcam, ab10099, Polyclonal Antibody):
https://www.abcam.com/products/primary-antibodies/vps35-antibody-ab10099.html
anti-β-amyloid (1–40) (Cell Signaling Technology, 12990, D8Q7I):
https://www.cellsignal.cn/products/primary-antibodies/b-amyloid-1-40-d8q7i-rabbit-mab/12990
anti-β-amyloid (1–42) (Cell Signaling Technology, 14974, D9A3A):
https://www.cellsignal.cn/products/primary-antibodies/b-amyloid-1-42-d9a3a-rabbit-mab/14974
anti-TGN46 (Invitrogen, MA3-063, 2F7.1):
https://www.thermofisher.cn/cn/zh/antibody/product/TGN46-Antibody-clone-2F7-1-Monoclonal/MA3-063
anti-VDAC (Cell Signaling Technology, 4661S, D73D12):
https://www.cellsignal.cn/products/primary-antibodies/vdac-d73d12-rabbit-mab/4661
anti-Rab5 (Cell Signaling Technology, 3547, C8B1):
https://www.cellsignal.cn/products/primary-antibodies/rab5-c8b1-rabbit-mab/3547
anti-LAMP2 (Proteintech, 27823-1-AP, Polyclonal Antibody):
https://www.ptglab.com/products/LAMP2-Antibody-27823-1-AP.htm
anti-RCAS1 (Cell Signaling Technology, 12290S, D2B6N):
https://www.cellsignal.cn/products/primary-antibodies/rcas1-d2b6n-xp-rabbit-mab/12290
anti-CANX (Cell Signaling Technology, 2679, C5C9):
https://www.cellsignal.cn/products/primary-antibodies/calnexin-c5c9-rabbit-mab/2679
anti-tau (Cell Signaling Technology, 46687, D1M9X):
https://www.cellsignal.cn/products/primary-antibodies/tau-d1m9x-xp-rabbit-mab/46687
anti-phospho-S396-tau (Abcam, ab32057, E178):
https://www.abcam.com/products/primary-antibodies/tau-phospho-s396-antibody-e178-ab32057.html
anti-α-tubulin (GeneTex, GTX628802, GT114):
https://www.genetex.cn/Product/Detail/alpha-Tubulin-antibody-GT114/GTX628802
anti-SorLA/SORL1 antibody (Abcam, ab190684, EPR14670):
https://www.abcam.com/products/primary-antibodies/sorlasorl1-antibody-epr14670-ab190684.html
goat anti-rabbit HRP-conjugated secondary antibody (ZSGB-BIO, ZB-2301, Polyclonal Antibody):
http://www.zsbio.com/product/ZB-2301
goat anti-mouse HRP-conjugated secondary antibody (ZSGB-BIO, ZB-2305, Polyclonal Antibody):
http://www.zsbio.com/product/ZB-2305
rabbit anti-goat HRP-conjugated secondary antibody (ZSGB-BIO, ZB-2306, Polyclonal Antibody):
http://www.zsbio.com/product/ZB-2306
anti-Iba1 (Wako, 019-19741, Polyclonal Antibody):
https://labchem-wako.fujifilm.com/us/product/detail/W01W0101-1974.html
Alexa FluorTM 488 goat anti-rabbit IgG (Invitrogen, A-11034, Polyclonal Antibody):
https://www.thermofisher.cn/cn/zh/antibody/product/Goat-anti-Rabbit-IgG-H-L-Highly-Cross-Adsorbed-Secondary-Antibody-Polyclonal/A-11034
Alexa FluorTM 594 goat anti-rabbit IgG (Invitrogen, A-11037, Polyclonal Antibody):

https://www.thermofisher.cn/cn/zh/antibody/product/Goat-anti-Rabbit-IgG-H-L-Highly-Cross-Adsorbed-Secondary-Antibody-Polyclonal/A-11037
Alexa FluorTM Plus 488 goat anti-mouse IgG (Invitrogen, A-32723, Polyclonal Antibody):
https://www.thermofisher.cn/cn/zh/antibody/product/Goat-anti-Mouse-IgG-H-L-Highly-Cross-Adsorbed-Secondary-Antibody-Polyclonal/A32723
Alexa FluorTM 594 goat anti-mouse IgG (Invitrogen, A-11032, Polyclonal Antibody):
https://www.thermofisher.cn/cn/zh/antibody/product/Goat-anti-Mouse-IgG-H-L-Highly-Cross-Adsorbed-Secondary-Antibody-Polyclonal/A-11032
4’,6-diamidino-2-phenylindole (DAPI, Solarbio, C0065):
https://www.solarbio.com/goods-258.html
poly horseradish peroxidase anti-rabbit secondary antibody (ZSGB-BIO, PV-9001):
http://www.zsbio.com/product/PV-9001
poly horseradish peroxidase anti-mouse secondary antibody (ZSGB-BIO, PV-9002):
http://www.zsbio.com/product/PV-9002
FITC Annexin V Apoptosis Detection Kit with PI (Biolegend, 640914):
https://www.biolegend.com/de-at/explore-new-products/fitc-annexin-v-apoptosis-detection-kit-with-pi-8230

Eukaryotic cell lines

Policy information about cell lines and Sex and Gender in Research

Cell line source(s)	SH-SY5Y, HEK293T, and BV2 were obtained from the Cell Resource Center, Pecking Union Medical College. N2a-APP, was generous gifted by Prof. B. H. Xu (Shenzhen Center of Disease Control and Prevention, China).
Authentication	None of the cell lines used were authenticated.
Mycoplasma contamination	All cell lines tested negative for mycoplasma contamination.
Commonly misidentified lines (See ICLAC register)	None commonly misidentified cell lines were used.

Palaeontology and Archaeology

Specimen provenance	*Provide provenance information for specimens and describe permits that were obtained for the work (including the name of the issuing authority, the date of issue, and any identifying information). Permits should encompass collection and, where applicable, export.*
Specimen deposition	*Indicate where the specimens have been deposited to permit free access by other researchers.*
Dating methods	*If new dates are provided, describe how they were obtained (e.g. collection, storage, sample pretreatment and measurement), where they were obtained (i.e. lab name), the calibration program and the protocol for quality assurance OR state that no new dates are provided.*

☐ Tick this box to confirm that the raw and calibrated dates are available in the paper or in Supplementary Information.

Ethics oversight	*Identify the organization(s) that approved or provided guidance on the study protocol, OR state that no ethical approval or guidance was required and explain why not.*

Note that full information on the approval of the study protocol must also be provided in the manuscript.

Animals and other research organisms

Policy information about studies involving animals; ARRIVE guidelines recommended for reporting animal research, and Sex and Gender in Research

Laboratory animals	APP/PS1 mice (n=4) and C57BL/6 mice (n=4), males, aged 7 months at time of detection, were used in this study. ALDH2 KO mice (n=5) and the control C57BL/6 mice (n=4), males, aged 3 months at time of detection, were used in this study. Mice were maintained on a reversed 12 h:12 h light:dark cycle at constant temperature (22 ± 1°C) and humidity with free access to food and water. Taking into consideration the exacerbating effect of the rs671 AA genotype on A score in male individuals, and the equal effects (higher Aβ deposits and Aβ40/42 ratio) on males and females, we used male APP/PS1 mice and male ALDH2 knockout (KO) mice in our animal experiments.
Wild animals	The study did not involve wild animals.
Reporting on sex	The finding in this study applied to male mice.
Field-collected samples	The study did not involve samples collected from the field.

Ethics oversight | All animal experiments were performed in accordance with a protocol approved by the institutional Animal Care and Use Committee of Institute of Basic Medical Sciences.

Note that full information on the approval of the study protocol must also be provided in the manuscript.

Clinical data

Policy information about clinical studies

All manuscripts should comply with the ICMJEguidelines for publication of clinical research and a completedCONSORT checklist must be included with all submissions.

Clinical trial registration | *Provide the trial registration number from ClinicalTrials.gov or an equivalent agency.*

Study protocol | *Note where the full trial protocol can be accessed OR if not available, explain why.*

Data collection | *Describe the settings and locales of data collection, noting the time periods of recruitment and data collection.*

Outcomes | *Describe how you pre-defined primary and secondary outcome measures and how you assessed these measures.*

Dual use research of concern

Policy information about dual use research of concern

Hazards

Could the accidental, deliberate or reckless misuse of agents or technologies generated in the work, or the application of information presented in the manuscript, pose a threat to:

No	Yes	
☐	☐	Public health
☐	☐	National security
☐	☐	Crops and/or livestock
☐	☐	Ecosystems
☐	☐	Any other significant area

Experiments of concern

Does the work involve any of these experiments of concern:

No	Yes	
☐	☐	Demonstrate how to render a vaccine ineffective
☐	☐	Confer resistance to therapeutically useful antibiotics or antiviral agents
☐	☐	Enhance the virulence of a pathogen or render a nonpathogen virulent
☐	☐	Increase transmissibility of a pathogen
☐	☐	Alter the host range of a pathogen
☐	☐	Enable evasion of diagnostic/detection modalities
☐	☐	Enable the weaponization of a biological agent or toxin
☐	☐	Any other potentially harmful combination of experiments and agents

Plants

Seed stocks | *Report on the source of all seed stocks or other plant material used. If applicable, state the seed stock centre and catalogue number. If plant specimens were collected from the field, describe the collection location, date and sampling procedures.*

Novel plant genotypes | *Describe the methods by which all novel plant genotypes were produced. This includes those generated by transgenic approaches, gene editing, chemical/radiation-based mutagenesis and hybridization. For transgenic lines, describe the transformation method, the number of independent lines analyzed and the generation upon which experiments were performed. For gene-edited lines, describe the editor used, the endogenous sequence targeted for editing, the targeting guide RNA sequence (if applicable) and how the editor was applied.*

Authentication | *Describe any authentication procedures for each seed stock used or novel genotype generated. Describe any experiments used to assess the effect of a mutation and, where applicable, how potential secondary effects (e.g. second site T-DNA insertions, mosiacism, off-target gene editing) were examined.*

ChIP-seq

Data deposition

- [ ] Confirm that both raw and final processed data have been deposited in a public database such as GEO.
- [ ] Confirm that you have deposited or provided access to graph files (e.g. BED files) for the called peaks.

Data access links<br>*May remain private before publication.*	*For "Initial submission" or "Revised version" documents, provide reviewer access links. For your "Final submission" document, provide a link to the deposited data.*
Files in database submission	*Provide a list of all files available in the database submission.*
Genome browser session<br>(e.g. UCSC)	*Provide a link to an anonymized genome browser session for "Initial submission" and "Revised version" documents only, to enable peer review. Write "no longer applicable" for "Final submission" documents.*

Methodology

Replicates	*Describe the experimental replicates, specifying number, type and replicate agreement.*
Sequencing depth	*Describe the sequencing depth for each experiment, providing the total number of reads, uniquely mapped reads, length of reads and whether they were paired- or single-end.*
Antibodies	*Describe the antibodies used for the ChIP-seq experiments; as applicable, provide supplier name, catalog number, clone name, and lot number.*
Peak calling parameters	*Specify the command line program and parameters used for read mapping and peak calling, including the ChIP, control and index files used.*
Data quality	*Describe the methods used to ensure data quality in full detail, including how many peaks are at FDR 5% and above 5-fold enrichment.*
Software	*Describe the software used to collect and analyze the ChIP-seq data. For custom code that has been deposited into a community repository, provide accession details.*

Flow Cytometry

Plots

Confirm that:

- [x] The axis labels state the marker and fluorochrome used (e.g. CD4-FITC).
- [x] The axis scales are clearly visible. Include numbers along axes only for bottom left plot of group (a 'group' is an analysis of identical markers).
- [ ] All plots are contour plots with outliers or pseudocolor plots.
- [x] A numerical value for number of cells or percentage (with statistics) is provided.

Methodology

Sample preparation	A total of 1×106 SH-SY5Y cells in a 6-cm dish were treated with 2 µM (±)-4-HNE, 2 µM (S)-4-HNE, or 2 µM (R)-4-HNE for 72 h. Following the treatment, the cells were washed with PBS, harvested, and labeled using the FITC Annexin V Apoptosis Detection Kit with PI (Biolegend, 640914), following the manufacturer's instructions.
Instrument	CytoFLEX (Beckman, USA)
Software	CytExpert software (v2.4.028)
Cell population abundance	The gated singlets were classified using Annexin V-FITC and PI signals and represented as dot plots. The percentage of apoptotic cells was determined by combining the percentages of early apoptotic cells (Annexin V high, PI low) and late apoptotic/necrotic cells (Annexin V high, PI high)
Gating strategy	The initial gating of the whole cell population, excluding the debris, was based on FCS-A/SSC-A.

- [x] Tick this box to confirm that a figure exemplifying the gating strategy is provided in the Supplementary Information.

Magnetic resonance imaging

Experimental design

Design type — *Indicate task or resting state; event-related or block design.*

Design specifications — *Specify the number of blocks, trials or experimental units per session and/or subject, and specify the length of each trial or block (if trials are blocked) and interval between trials.*

Behavioral performance measures — *State number and/or type of variables recorded (e.g. correct button press, response time) and what statistics were used to establish that the subjects were performing the task as expected (e.g. mean, range, and/or standard deviation across subjects).*

Acquisition

Imaging type(s) — *Specify: functional, structural, diffusion, perfusion.*

Field strength — *Specify in Tesla*

Sequence & imaging parameters — *Specify the pulse sequence type (gradient echo, spin echo, etc.), imaging type (EPI, spiral, etc.), field of view, matrix size, slice thickness, orientation and TE/TR/flip angle.*

Area of acquisition — *State whether a whole brain scan was used OR define the area of acquisition, describing how the region was determined.*

Diffusion MRI ☐ Used ☐ Not used

Preprocessing

Preprocessing software — *Provide detail on software version and revision number and on specific parameters (model/functions, brain extraction, segmentation, smoothing kernel size, etc.).*

Normalization — *If data were normalized/standardized, describe the approach(es): specify linear or non-linear and define image types used for transformation OR indicate that data were not normalized and explain rationale for lack of normalization.*

Normalization template — *Describe the template used for normalization/transformation, specifying subject space or group standardized space (e.g. original Talairach, MNI305, ICBM152) OR indicate that the data were not normalized.*

Noise and artifact removal — *Describe your procedure(s) for artifact and structured noise removal, specifying motion parameters, tissue signals and physiological signals (heart rate, respiration).*

Volume censoring — *Define your software and/or method and criteria for volume censoring, and state the extent of such censoring.*

Statistical modeling & inference

Model type and settings — *Specify type (mass univariate, multivariate, RSA, predictive, etc.) and describe essential details of the model at the first and second levels (e.g. fixed, random or mixed effects; drift or auto-correlation).*

Effect(s) tested — *Define precise effect in terms of the task or stimulus conditions instead of psychological concepts and indicate whether ANOVA or factorial designs were used.*

Specify type of analysis: ☐ Whole brain ☐ ROI-based ☐ Both

Statistic type for inference — *Specify voxel-wise or cluster-wise and report all relevant parameters for cluster-wise methods.*

(See Eklund et al. 2016)

Correction — *Describe the type of correction and how it is obtained for multiple comparisons (e.g. FWE, FDR, permutation or Monte Carlo).*

Models & analysis

n/a	Involved in the study
☐	☐ Functional and/or effective connectivity
☐	☐ Graph analysis
☐	☐ Multivariate modeling or predictive analysis

Functional and/or effective connectivity	*Report the measures of dependence used and the model details (e.g. Pearson correlation, partial correlation, mutual information).*
Graph analysis	*Report the dependent variable and connectivity measure, specifying weighted graph or binarized graph, subject- or group-level, and the global and/or node summaries used (e.g. clustering coefficient, efficiency, etc.).*
Multivariate modeling and predictive analysis	*Specify independent variables, features extraction and dimension reduction, model, training and evaluation metrics.*
